# Residential mobility and receipt of measles, mumps and rubella vaccination: analysis of linked primary care electronic health records in a London region

**DOI:** 10.23889/ijpds.v11i1.2963

**Published:** 2026-03-25

**Authors:** Nicola Firman, Laura North, Milena Marszalek, Marta Wilk, Ana Gutierrez, Rhodri Johnson, Carol Dezateux

**Affiliations:** 1 Centre for Primary Care, Wolfson Institute of Population Health, Barts and the London School of Medicine and Dentistry, Queen Mary University of London, Yvonne Carter Building, 58 Turner Street, London, E1 2AB; 2 Swansea University Medical School, Faculty of Medicine, Health & Life Sciences, Singleton Park, Swansea, SA2 8PP

**Keywords:** MMR, vaccination, children, residential mobility, electronic health records

## Abstract

**Background:**

Residential mobility in early life may disrupt access to health care. We examined associations between residential mobility in the first two years of life and receipt of first measles, mumps and rubella (MMR) vaccination by 24 months of age.

**Methods:**

We analysed electronic health records for children born between 01/01/2014 and 30/10/2019 and registered with primary care general practices (GPs) in north-east London (NEL). Primary outcome was receipt of first MMR vaccination between 12 and 24 months of age and residential mobility was defined by number of GP-recorded addresses by vaccination date or 24 months of age. We used logistic regression to estimate the odds ratio (OR) and 95% confidence intervals (CI) of receipt of MMR vaccination by residential mobility, adjusting for sex, ethnicity, number of children in the household, household composition, area-level deprivation, and local government area of residence.

**Results:**

We included 150,949 children (51.0% boys) of whom 127,958 (84.8%) had received a first MMR vaccination and 22.3% had more than one GP-recorded address. Compared to children with one GP-recorded address, children with multiple GP-recorded addresses were at higher risk of not receiving their first MMR vaccination by 24 months of age. Those with two GP-recorded addresses had a 54% increased likelihood (OR: 0.46; 95% CI: 0.44,0.48), and those with three or more GP-recorded addresses a 68% increased likelihood (0.32; 95% CI: 0.29,0.36), compared to those with one.

**Conclusions:**

Children who change address in early life are less likely to be protected against measles and other infections. Measles outbreaks in the UK have been in urban areas with high residential mobility and low MMR vaccine uptake suggesting the need for review of the immunisation status of children newly registered with GPs.

## Introduction

Measles is one of the most contagious infectious diseases, with a reproduction number between 12 and 18 [1]. Measles, mumps, and rubella (MMR) vaccination is the most effective public health measure to prevent measles infection, and whilst children are eligible for the first dose of the vaccine at 12 months in the United Kingdom (UK) [2], the health service is failing to vaccinate children on time. Coverage for first MMR vaccination (MMR1) between 12 and 24 months of age in the UK is around 89% [3]. The north-east London (NEL) region has the lowest coverage in the UK at around 80% [3], well below the 95% recommended to achieve herd immunity. This low coverage is likely to have contributed to almost 2,563 laboratory confirmed cases of measles in England, the majority in London, in the first ten months of 2024 [4]. It has been hypothesised that children who move home frequently are less likely to receive vaccinations.

A child’s home environment may affect their health and development through a variety of factors; the social and economic stability of the environment, the quality of the physical space, and the household members sharing and shaping use of the environment [5–15]. A growing body of literature suggests that residential mobility during childhood may be associated with adverse health and educational outcomes throughout childhood, adolescence and into adulthood [16].

It has been shown that children who have moved home at least twice in the first year of life were more likely to attend hospital for ear, nose and throat infections, injuries, gastroenteritis, asthma, influenza, and dental conditions [8]. There is mixed evidence about the association between residential mobility and receipt of childhood vaccinations. Researchers found children in England were less likely to have received the MMR1 vaccination by three years of age, if they had moved home twice or more, compared to not moving, between the ages of nine months and three years [11]. Similarly, in Canada, the likelihood of being incompletely immunised by age seven was greater in children who had moved residence two times or more, compared to those who had moved one time or less [17]. Conversely, a study investigating childhood immunisation rates in Wales, UK showed no differences between children experiencing residential mobility or not [10].

Given these conflicting findings and the lack of studies focused in urban areas with high residential mobility, we examined associations between residential mobility in an ethnically diverse, disadvantaged population with low MMR1 vaccine uptake. We hypothesised that children experiencing changes in address in the first 24 months would be less likely to receive the MMR1 vaccination between 12 and 24 months of age, compared with those without residential mobility.

## Methods

### Study design and setting

We carried out a retrospective longitudinal study using primary care electronic health records (EHRs) from all general practices (GP) in seven geographically contiguous areas in NEL: Barking & Dagenham, City & Hackney, Havering, Newham, Redbridge, Tower Hamlets, and Waltham Forest. The study protocol can be found in supplementary file 1 and the Reporting of studies Conducted using Observational Routinely-collected health Data (RECORD) Statement in supplementary file 2.

### Study population

We defined the cohort as children registered with a NEL GP on their second birthday between 1^st^ January 2016 and 30^th^ October 2021. These children would have been eligible to receive their MMR1 vaccination between 12 and 24 months of age between 1^st^ January 2015 and 30^th^ October 2021. Children could enter the cohort at any point between birth and their second birthday.

### Data sources

Pseudonymised data were provided from the NEL Discovery Data Service which receives daily primary care EHR data from all GPs in NEL. Demographic and clinical data were extracted for children ever registered with a NEL GP and included children who may have died or left the area. Data were extracted on 23^rd^ November 2021 and included all clinical events up to 1^st^ November 2021. All data were extracted and managed according to UK National Health Service (NHS) information governance requirements [18].

### Data processing

Every addressable location in Great Britain is assigned a Unique Property Reference Number (UPRN). UPRNs identify a place of residence at a granular level, identifying individual properties, for example houses or flats within a block or building shell. UPRNs are allocated to GP-recorded addresses using the validated AddreSS MatchInG to Unique Property Reference Numbers (ASSIGN) algorithm [19], and pseudonymised into Residential Anonymised Linkage Fields (RALFs) within the Discovery Data Service, using a study-specific encryption key. We have previously described the process for identifying household members at a point in time [20, 21].

**Figure 1: Study sample:**
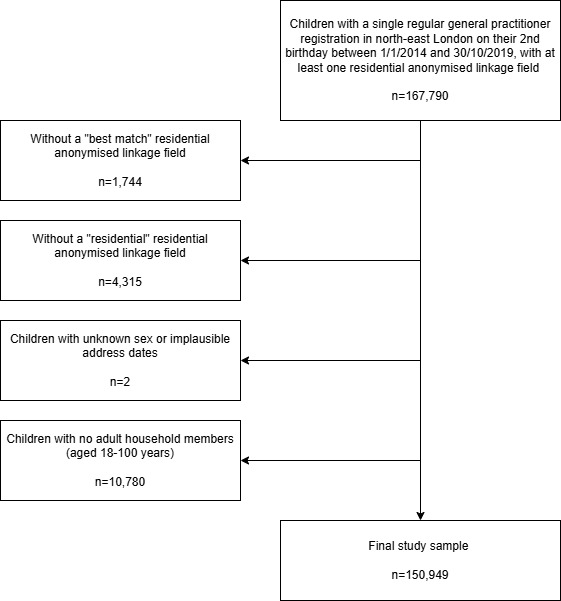


We identified 167,790 children born between 1^st^ January 2014 and 30^th^ October 2019 and with a single ‘regular’ (as opposed to temporary) NEL GP registration on their second birthday, with at least one RALF. We excluded 1,744 children with a poor-quality RALF match, and 4,315 with a RALF associated with a non-residential building. We further excluded 2 with unknown sex or implausible address dates. We retained only those living with at least one adult aged 18-100 years (figure 1). Our final study sample comprised 150,949 children (90.0%). Characteristics of those excluded from the study sample can be found in supplementary file 3 table 1.

We extracted sociodemographic, household and geographic data for each child, together with – for each child – all clinical events relating to MMR1 procedures. We derived a proxy date of birth by assigning day of birth to the first day of the week using the calendar week and year of birth.

### Primary outcome

We defined timely MMR1 vaccination as receipt of the MMR1 vaccination between 12 and 24 months of age. This is consistent with the Cover of Vaccination Evaluated Rapidly (COVER) measures in place during the study period [3]. We identified MMR1 vaccination using a pre-specified list of clinical Systematized Medical Nomenclature for Medicine (SNOMED) terms (supplementary file 3 table 2). In accordance with the UK Health Security Agency guidelines [2], MMR1 vaccinations given before 12 months of age were ignored, and in these analyses, follow-up was censored at 24 months, however some children may have received their vaccination after this age.

### Main exposure

**Figure 2: Counting general practice-recorded addresses as unique Residential Anonymised Linkage Fields (RALFs):**
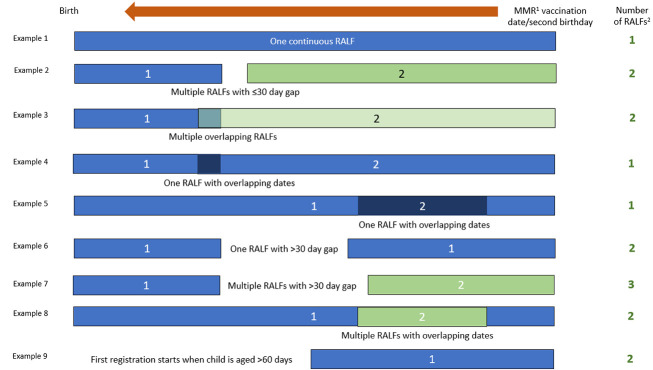
^1^Measles, mumps and rubella. ^2^Residential anonymised linkage fields.

The main exposure was residential mobility during early childhood, defined as the number of GP-recorded addresses in the child’s EHR, as identified by unique residential RALFs between their earliest GP registration and either their MMR1 vaccination date or second birthday. We assumed that children with periods of time without a NEL GP registration were living elsewhere and added one to the count of RALFs for each period of greater than 30 days when a child was not registered with a NEL GP. Similarly, we added one to the count of RALFs for children who were aged greater than 60 days old when they first registered with a NEL GP. Residential mobility (number of RALFs) was categorised into one, two, three or more (see figure 2). 2,345 (1.6%) of 150,949 children had at least one unregistered period lasting more than 30 days (2,294 had one unregistered period, 51 had two or more). Unregistered periods of more than 30 days ranged from 31 to 657 days, with a mean length of 113.5 days (standard deviation: 100.7) and median length of 74 days (interquartile range: 44,146). We assume that 24,943 (16.5%) of 150,949 children moved into NEL during the period of follow-up – they were aged more than 60 days old at the time of their first NEL GP registration. The age at first GP registration ranged from 0 to 778 days, with a mean age of 226.4 days (standard deviation: 171.9) and a median age of 169 days (interquartile range: 78,336).

### Covariates

We included individual-, household-, and area-level characteri-stics as covariates. Individual-level characteristics included the child’s sex and ethnic background, classified using the NHS 16+1 categorisation: White British, White Irish, Other White, Chinese, White & Asian, White & Black African, White & Black Caribbean, Other Asian, Other Mixed, Other, Bangladeshi, Indian, Pakistani, Black African, Black Caribbean, and Other Black [22].

Household-level characteristics included the number of children living in the household and household composition. Both were defined on the date of the child’s second birthday. The number of children in the household was defined as the total number of people (including the cohort child) aged under 18 years sharing the same RALF as the cohort child on the cohort child’s second birthday. We categorised this into: one, two, three, four or more. We categorised household composition using a modified Harper and Mayhew method [23] by counting the number of household members in three age brackets: 0-18 years (children), 19-64 years (working age adults) and 65 or older (older adults) and grouping into: working-aged adults with children; a single working age adult with children; other.

Area-level characteristics included local authority of the child’s GP and area-level deprivation. We used the 2019 Income Deprivation Affecting Children Index (IDACI) at decile level [24], linked using the 2011 Lower layer Super Output Area (LSOA) of the child’s home address. An LSOA has an average population of 1,500 people or 650 households. The IDACI score measures the proportion of children under 16 in low-income households for an area. IDACI deciles were collapsed into quintiles from most to least deprived.

### Statistical analyses

We explored variation in the proportion of children receiving a MMR1 vaccination by residential mobility and other covariates, and described the differences in the proportion of children receiving the MMR1 vaccination by 24 months of age. We also explored variation in residential mobility by the covariates. We conducted binary logistic regression to estimate the crude and adjusted odds (odds ratio [OR] and 95% confidence interval [CI]) of a timely MMR1 vaccination by residential mobility, using a stepwise approach to adjust for individual-, household-, and area-level covariates. We present OR (95% CI) of a timely MMR1 vaccination in four models: 1) Univariable; 2) Adjustment for individual-level factors (sex and ethnic background); 3) Adjustment for individual- and household-level factors (number of children in the household and household composition); 4) Adjustment for individual-, household-, and area-level factors (IDACI quintile and local authority).

All analyses were conducted using Stata (MP/17.0).

### Sensitivity analyses

We repeated all analyses without adding one more to the count of addresses for children who were aged greater than 60 days old when they first registered with a NEL GP (figure 2 example 9) or for each period of greater than 30 days when a child was not registered with a NEL GP (figure 2 examples 6 and 7). We also repeated all analyses on a sub-sample of 109,711 children born between 1^st^ January 2014 and 20_th_ March 2018 and therefore eligible to receive their MMR1 vaccination between 12 and 24 months of age between 1^st^ January 2015 and 20^th^ March 2020. The receipt of MMR1 vaccination among these children is expected to be unaffected by the 2019 Coronavirus (COVID-19) pandemic. Results of both sensitivity analyses are presented in the supplementary material.

### Patient and public involvement

Patients and the public were not involved in the design of this research.

## Results

Our sample of 150,949 children (51.0% boys) was ethnically diverse (30.8% White [White British, White Irish and Other White], 8.2% Black [Black African, Black Caribbean and Other Black], 21.4% South Asian [Bangladeshi, Indian and Pakistani]; table 1). Overall, 127,958 of 150,949 (84.8%; 95% CI: 84.6,84.9) children received their MMR1 vaccination between 12 and 24 months of age. The majority (*n*=117,156; 77.6%) of children had only one GP-recorded address before either their MMR1 vaccination or second birthday, 21.0% (*n* = 31,769) had two, and 1.3% (*n* = 2,024) had three or more (the complete distribution of changes in GP-recorded address can be found in supplementary file 3 table 3).

**Table 1:** Sample characteristics

	**All children**		
	**n**	**%**	**95% CI^1^**
**MMR^2^ status**			
No MMR between 12 and 24 months	22991	15.2	15.1,15.4
MMR between 12 and 24 months	127958	84.8	84.6,84.9
**Number of GP-recorded addresses^3^**			
1	117156	77.6	77.4,77.8
2	31769	21.0	20.8,21.3
3 or more	2024	1.4	1.3,1.4
**Sex**			
Male	77036	51.0	50.8,51.3
Female	73913	49.0	48.7,49.2
**Ethnic background**			
White British	27492	18.2	18.0,18.4
White Irish	324	0.2	0.2,0.2
Other White	18617	12.3	12.2,12.5
Chinese	936	0.6	0.6,0.7
White & Asian	1533	1.0	1.0,1.1
White & Black African	1142	0.8	0.7,0.8
White & Black Caribbean	1157	0.8	0.7,0.8
Other Asian	4497	3.0	2.9,3.1
Other Mixed	3206	2.1	2.1,2.2
Other	6442	4.3	4.2,4.4
Bangladeshi	14350	9.5	9.3,9.6
Indian	8062	5.3	5.2,5.5
Pakistani	9878	6.5	6.4,6.7
Black African	7130	4.7	4.6,4.8
Black Caribbean	1421	0.9	0.9,1.0
Other Black	3800	2.5	2.4,2.6
Missing	40962	27.1	26.9,27.4
**Number of children in the household^4^**			
1	48049	31.8	31.6,32.1
2	48020	31.8	31.6,32.1
3	27978	18.5	18.3,18.7
4 or more	26902	17.8	17.6,18.0
**Household composition^5^**			
Adults with child(ren)	111529	73.9	73.7,74.1
Single adult with child(ren)	25253	16.7	16.5,16.9
Other	14167	9.4	9.2,9.5
**IDACI quintile^6^**			
1 - most deprived	61779	40.9	40.7,41.2
2	56157	37.2	37.0,37.4
3	21491	14.2	14.1,14.4
4	8300	5.5	5.4,5.6
5 - least deprived	3191	2.1	2.0,2.2
Missing	31	0.0	0.0,0.0
**Local authority**			
Barking	19376	12.8	12.7,13.0
City & Hackney	19589	13.0	12.8,13.1
Havering	18264	12.1	12.0,12.3
Newham	26315	17.4	17.2,17.6
Redbridge	24162	16.0	15.8,16.2
Tower Hamlets	21029	13.9	13.7,14.1
Waltham Forest	22214	14.7	14.5,14.9

^1^Confidence interval. ^2^Measles, mumps and rubella vaccination status. ^3^General practice. ^4^Number of people aged 0-17.9 years sharing the same address on the date of the child’s second birthday. ^5^Household composition defined on the date of the child’s second birthday. ^6^2019 Income Deprivation Affecting Children Index quintile.

Almost one third of children were single-children (31.8%), and most (73.9%) lived with at least two working age adults. The majority (78.1%) lived in areas in the two most deprived IDACI quintiles.

The proportion receiving a MMR1 vaccination by 24 months of age varied by residential mobility (table 2). The proportion of children with two (75.5%; 95% CI: 75.0,76.0) or three or more GP-recorded addresses (68.7%; 66.7,70.7) were less likely to have received their MMR1 vaccination by their second birthday, compared with those with one GP-recorded address (87.6%; 87.4,87.7). The proportion of children with a MMR1 vaccination by 24 months of age was higher than average among children from White British, Chinese, White & Asian, Other Asian, and South Asian ethnic backgrounds, and lower than average among those from White Irish, Other White, White & Black Caribbean, Other, and Black ethnic backgrounds. The proportion vaccinated by 24 months was also higher than average among children living in households with one or two children, and lower among those living with a single adult. Across the seven local authorities, the proportion of children receiving their MMR1 vaccination by 24 months of age ranged from 75.8% (City & Hackney) to 89.9% (Havering and Tower Hamlets).

**Table 2:** Sample characteristics by first measles, mumps and rubella (MMR) vaccination status

	**No MMR between 12 and 24 months**			**MMR between 12 and 24 months**		
	**n**	**%**	**95% CI^1^**	**n**	**%**	**95% CI^1^**
**All**	22991	15.2	15.1,15.4	127958	84.8	84.6,84.9
**Number of GP-recorded addresses^2^**						
1	14581	12.4	12.3,12.6	102575	87.6	87.4,87.7
2	7777	24.5	24.0,25.0	23992	75.5	75.0,76.0
3 or more	633	31.3	29.3,33.3	1391	68.7	66.7,70.7
**Sex**						
Male	11890	15.4	15.2,15.7	65146	84.6	84.3,84.8
Female	11101	15.0	14.8,15.3	62812	85.0	84.7,85.2
**Ethnic background**						
White British	2873	10.5	10.1,10.8	24619	89.5	89.2,89.9
White Irish	78	24.1	19.7,29.0	246	75.9	71.0,80.3
Other White	3916	21.0	20.5,21.6	14701	79.0	78.4,79.5
Chinese	74	7.9	6.3,9.8	862	92.1	90.2,93.7
White & Asian	167	10.9	9.4,12.6	1366	89.1	87.4,90.6
White & Black African	166	14.5	12.6,16.7	976	85.5	83.3,87.4
White & Black Caribbean	234	20.2	18.0,22.6	923	79.8	77.4,82.0
Other Asian	478	10.6	9.8,11.6	4019	89.4	88.4,90.2
Other Mixed	512	16.0	14.7,17.3	2694	84.0	82.7,85.3
Other	1773	27.5	26.4,28.6	4669	72.5	71.4,73.6
Bangladeshi	1271	8.9	8.4,9.3	13079	91.1	90.7,91.6
Indian	927	11.5	10.8,12.2	7135	88.5	87.8,89.2
Pakistani	1153	11.7	11.1,12.3	8725	88.3	87.7,88.9
Black African	1111	15.6	14.8,16.4	6019	84.4	83.6,85.2
Black Caribbean	388	27.3	25.1,29.7	1033	72.7	70.3,74.9
Other Black	802	21.1	19.8,22.4	2998	78.9	77.6,80.2
Missing	7068	17.3	16.9,17.6	33894	82.7	82.4,83.1
**Number of children in the household^3^**						
1	5828	12.1	11.8,12.4	42221	87.9	87.6,88.2
2	6530	13.6	13.3,13.9	41490	86.4	86.1,86.7
3	4483	16.0	15.6,16.5	23495	84.0	83.5,84.4
4 or more	6150	22.9	22.4,23.4	20752	77.1	76.6,77.6
**Household composition^4^**						
Adults with child(ren)	16540	14.8	14.6,15.0	94989	85.2	85.0,85.4
Single adult with child(ren)	4397	17.4	16.9,17.9	20856	82.6	82.1,83.1
Other	2054	14.5	13.9,15.1	12113	85.5	84.9,86.1
**IDACI quintile^5^**						
1 - most deprived	9165	14.8	14.6,15.1	52614	85.2	84.9,85.4
2	9277	16.5	16.2,16.8	46880	83.5	83.2,83.8
3	3091	14.4	13.9,14.9	18400	85.6	85.1,86.1
4	1197	14.4	13.7,15.2	7103	85.6	84.8,86.3
5 - least deprived	251	7.9	7.0,8.9	2940	92.1	91.1,93.0
Missing	10	32.3	18.3,50.3	21	67.7	49.7,81.7
**Local authority**						
Barking	3244	16.7	16.2,17.3	16132	83.3	82.7,83.8
City & Hackney	4743	24.2	23.6,24.8	14846	75.8	75.2,76.4
Havering	1851	10.1	9.7,10.6	16413	89.9	89.4,90.3
Newham	3729	14.2	13.8,14.6	22586	85.8	85.4,86.2
Redbridge	3921	16.2	15.8,16.7	20241	83.8	83.3,84.2
Tower Hamlets	2132	10.1	9.7,10.6	18897	89.9	89.4,90.3
Waltham Forest	3371	15.2	14.7,15.7	18843	84.8	84.3,85.3

^1^Confidence interval. ^2^General practice. ^3^Number of people aged 0-17.9 years sharing the same address on the date of the child’s second birthday. ^4^Household composition defined on the date of the child’s second birthday. ^5^2019 Income Deprivation Affecting Children Index quintile.

The proportion of children with three or more GP-recorded addresses was higher than average among those from White Irish and Other White, Black African and Other Black ethnic backgrounds (table 3). Residential mobility also varied by the number of children in the household and household composition. The proportion of children with three or more GP-recorded addresses was higher among those living in households with four or more children or with a single adult.

**Table 3:** Sample characteristics by residential mobility (the number of general practice-recorded addresses)

	**One**			**Two**			**Three or more**		
	**n**	**%**	**95% CI^1^**	**n**	**%**	**95% CI^1^**	**n**	**%**	**95% CI^1^**
**All**	**117156**	**77.6**	**77.4,77.8**	**31769**	**21.1**	**20.8,21.3**	**2024**	**1.3**	**1.3,1.4**
**Sex**									
Male	59832	77.7	77.4,78.0	16176	21.0	20.7,21.3	1028	1.3	1.3,1.4
Female	57324	77.6	77.3,77.9	15593	21.1	20.8,21.4	996	1.3	1.3,1.4
**Ethnic background**									
White British	22143	80.5	80.1,81.0	5027	18.3	17.8,18.7	322	1.2	1.1,1.3
White Irish & Other White^2^	13695	72.3	71.7,72.9	4884	25.8	25.2,26.4	362	1.9	1.7,2.1
Chinese	696	74.4	71.5,77.1	227	24.3	21.6,27.1	13	1.4	0.8,2.4
White & Asian	1210	78.9	76.8,80.9	299	19.5	17.6,21.6	24	1.6	1.1,2.3
White & Black African	844	73.9	71.3,76.4	280	24.5	22.1,27.1	18	1.6	1.0,2.5
White & Black Caribbean	857	74.1	71.5,76.5	282	24.4	22.0,26.9	18	1.6	1.0,2.5
Other Asian	3586	79.7	78.5,80.9	857	19.1	17.9,20.2	54	1.2	0.9,1.6
Other Mixed	2411	75.2	73.7,76.7	738	23.0	21.6,24.5	57	1.8	1.4,2.3
Other	4630	71.9	70.8,73.0	1718	26.7	25.6,27.8	94	1.5	1.2,1.8
Bangladeshi	11385	79.3	78.7,80.0	2788	19.4	18.8,20.1	177	1.2	1.1,1.4
Indian	5873	72.8	71.9,73.8	2081	25.8	24.9,26.8	108	1.3	1.1,1.6
Pakistani	7721	78.2	77.3,79.0	2002	20.3	19.5,21.1	155	1.6	1.3,1.8
Black African	5336	74.8	73.8,75.8	1671	23.4	22.5,24.4	123	1.7	1.4,2.1
Black Caribbean	1062	74.7	72.4,76.9	337	23.7	21.6,26.0	22	1.5	1.0,2.3
Other Black	2837	74.7	73.3,76.0	891	23.4	22.1,24.8	72	1.9	1.5,2.4
Missing	32870	80.2	79.9,80.6	7687	18.8	18.4,19.1	405	1.0	0.9,1.1
**Number of children in the household^3^**									
1	37216	77.5	77.1,77.8	10195	21.2	20.9,21.6	638	1.3	1.2,1.4
2	37925	79.0	78.6,79.3	9503	19.8	19.4,20.1	592	1.2	1.1,1.3
3	21745	77.7	77.2,78.2	5860	20.9	20.5,21.4	373	1.3	1.2,1.5
4 or more	20270	75.3	74.8,75.9	6211	23.1	22.6,23.6	421	1.6	1.4,1.7
**Household composition^4^**									
Adults with child(ren)	86675	77.7	77.5,78.0	23424	21.0	20.8,21.2	1430	1.3	1.2,1.3
Single adult with child(ren)	18842	74.6	74.1,75.1	5943	23.5	23.0,24.1	468	1.9	1.7,2.0
Other	11639	82.2	81.5,82.8	2402	17.0	16.3,17.6	126	0.9	0.7,1.1
**IDACI quintile^5^**									
1 - most deprived	48016	77.7	77.4,78.0	12889	20.9	20.5,21.2	874	1.4	1.3,1.5
2	43154	76.8	76.5,77.2	12237	21.8	21.5,22.1	766	1.4	1.3,1.5
3	16726	77.8	77.3,78.4	4493	20.9	20.4,21.5	272	1.3	1.1,1.4
4	6578	79.3	78.4,80.1	1629	19.6	18.8,20.5	93	1.1	0.9,1.4
5 - least deprived	2665	83.5	82.2,84.8	507	15.9	14.7,17.2	19	0.6	0.4,0.9
Missing	17	54.8	37.4,71.1	14	45.2	28.9,62.6	0	0.0	
**Local authority**									
Barking	14198	73.3	72.6,73.9	4900	25.3	24.7,25.9	278	1.4	1.3,1.6
City & Hackney	15426	78.7	78.2,79.3	3940	20.1	19.6,20.7	223	1.1	1.0,1.3
Havering	13792	75.5	74.9,76.1	4224	23.1	22.5,23.7	248	1.4	1.2,1.5
Newham	20403	77.5	77.0,78.0	5539	21.0	20.6,21.5	373	1.4	1.3,1.6
Redbridge	18640	77.1	76.6,77.7	5155	21.3	20.8,21.9	367	1.5	1.4,1.7
Tower Hamlets	16877	80.3	79.7,80.8	3879	18.4	17.9,19.0	273	1.3	1.2,1.5
Waltham Forest	17820	80.2	79.7,80.7	4132	18.6	18.1,19.1	262	1.2	1.0,1.3

^1^Confidence interval. ^2^White Irish and Other White combined due to small cells. ^3^Number of people aged 0-17.9 years sharing the same address on the date of the child’s second birthday. ^4^Household composition defined on the date of the child’s second birthday. Three generation and skipped generation households combined with other household compositions due to small cell sizes. ^5^2019 Income Deprivation Affecting Children Index quintile. Missing category not reported due to small cell sizes.

**Figure 3: Odds ratios for measles, mumps and rubella vaccination by number of GP-recorded addresses, by 24 months of age:**
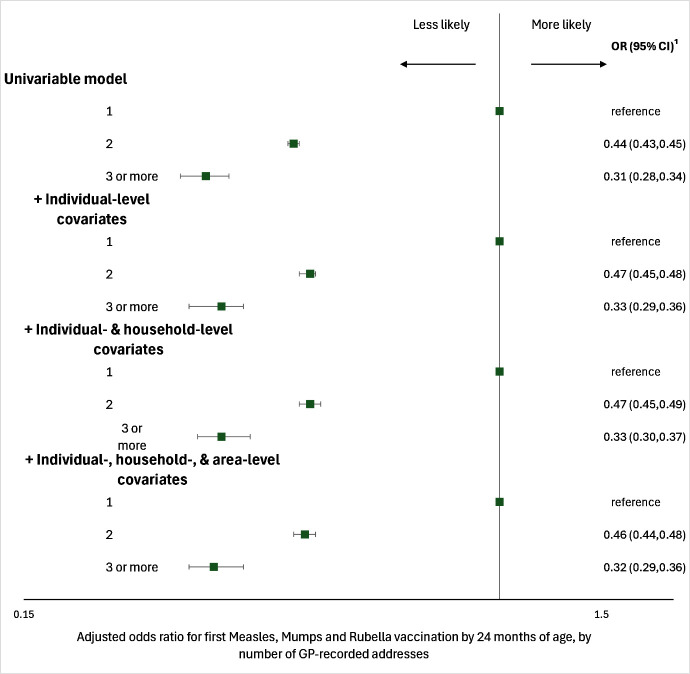
^1^Odds ratio (95% confidence interval).

In univariable analyses, children with two (OR: 0.44; 95% CI: 0.43,0.45) or three or more GP-recorded addresses (0.31; 0.28,0.34) were less likely than those with only one to receive their MMR1 vaccination by 24 months of age (figure 3 and supplementary file 3 table 4). In each iteration of the stepwise modelling process, adding individual-, household-, and area-level covariates marginally attenuated this association. In the fourth model (adjusting for individual-, household-, and area-level covariates) the likelihood of receiving a MMR1 vaccination by 24 months of age was 54% lower among (compared to 56% in univariable analyses) those with two GP-recorded addresses, and 68% lower among those with three (compared to 69% in univariable analyses), compared to those with only one GP-recorded address.

### Sensitivity analyses

In sensitivity analyses not adjusting for periods without a GP registration, 94.4% of children had only one GP-recorded address before their MMR1 vaccination or second birthday, 5.4% had two, and 0.2% three or more (supplementary file 3 table 5). The proportion of children receiving their MMR1 vaccination by 24 months of age varied by residential mobility (supplementary file 3 table 6). Children who had two, or three or more GP-recorded addresses were less likely to have received their MMR1 vaccination by their second birthday, compared with those with one (81.3%; 95% CI: 80.5,82.1; 74.5%; 68.9,79.4; 85.0%; 84.8,85.2; respectively). After adjustment for individual-, household-, and area-level covariates, children with two, or three or GP-recorded addresses were less likely than those with only one to receive their MMR1 vaccination by 24 months of age more (OR: 0.74; 95% CI: 0.69,0.79; 0.45; 0.33,0.62) (figure 4 and supplementary file 3 table 7).

**Figure 4: Odds ratios for measles, mumps and rubella vaccination by number of GP-recorded addresses, by 24 months of age, showing main analyses and results from two sensitivity analyses: 1) no adjustment for unregistered periods; 2) pre 2019 Coronavirus sub-sample:**
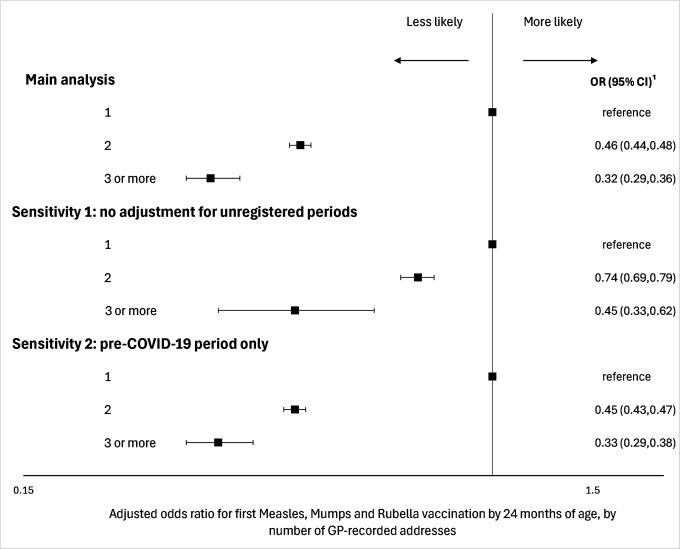
^1^Odds ratio (95% confidence interval).

Results were similar in sensitivity analyses restricted to children eligible for their MMR1 vaccination before the COVID-19 pandemic (supplementary file 3 tables 8-10). After adjustment for individual-, household-, and area-level covariates, children who had two, or three or more GP-recorded addresses were less likely than those with only one to receive their MMR1 vaccination by 24 months of age (OR: 0.45; 95% CI: 0.43,0.47; 0.33; 0.29,0.38) (figure 4 and supplementary file 3 table 10).

## Discussion

### Summary of key findings

To our knowledge, this is the first study in an urban, mobile, and ethnically diverse population to examine MMR1 receipt by residential mobility. After adjustment for covariates known to be associated with MMR1 vaccination and residential mobility, we found a reduced likelihood of MMR1 vaccination among children with increased residential mobility. Compared with children with just one address, children with two GP-recorded addresses were 54% less likely, and those with three or more, 68% less likely, to receive their MMR1 vaccination by 24 months of age.

### Strengths and limitations

We used routine primary care EHRs available for an entire population of children registered with all NHS general practices in one region of London. These results highlight inequalities in timely vaccination coverage, leaving children who experience residential mobility more susceptible to measles infection.

Coding of routine childhood vaccinations by primary care teams in NEL is facilitated by data entry templates with standardised coding enabling high quality recording of childhood vaccinations at the point of care. We developed and validated a SNOMED code specification which we mapped to Read code [25] and local system codes to ensure a maximally sensitive search strategy for identifying MMR1 vaccinations. We used robust statistical methods, including retrospective longitudinal analyses, to investigate the association between residential mobility and receipt of MMR1 vaccination. We confirmed our results were not impacted by the COVID-19 pandemic in our sensitivity analyses.

We used a robust methodology to identify household members on the second birthday. The ASSIGN algorithm has been shown to match 98.6% of primary care patient addresses to UPRNs [19]. We observed a marginally lower proportion of children receiving the MMR1 vaccination between 12 and 24 months of age among those excluded from the study sample, compared with those included. The explanation for this remains unclear. We excluded children living in non-residential households. It is possible that we included household members who no longer live at their registered address due to a time lag between a patients’ GP registrations, and a period of time where a patient has moved on from an area but remains registered with a GP. Hence, we may have overestimated the true number of children in the household. On the other hand, it is also possible we excluded children where it appeared they did not live with any other adults/household members in cases where other household members were not registered with a GP at the time of the child’s second birthday.

Our cohort definition included children registered with a NEL GP on their second birthday. By definition, this excluded children who may have been registered with and vaccinated by a NEL GP between 12 and 24 months of age, but who subsequently moved out of NEL and were not captured in our study sample, consequently excluding children experiencing residential mobility from our sample. Whilst not perfect, this approach enabled comparison of the characteristics of children registered with a NEL GP on their second birthday who did or did not receive MMR1 between 12 and 24 months of age. Additionally, we excluded children with multiple GP registrations, and those not living with any adults. It is possible these children have a different experience of MMR1 vaccination, compared with children included in our study sample.

We made some assumptions about residential mobility during periods of time when children were not registered with a NEL GP. We added one to the count of addresses for children aged greater than 60 days old when their first GP registration started, assuming these children had moved into NEL from elsewhere. Similarly, we added one to the count of addresses for each period of more than 30 days when a child was not registered with a NEL GP between their first registration and second birthday/date of MMR1 vaccination. We did not have information about the child’s place(s) of residence during these time periods and have assumed they lived elsewhere. We conducted sensitivity analyses without this adjustment, as we recognise it is possible unregistered periods are a result of administrative delays in registration. Whilst the magnitude of the effect of residential mobility was not as great, the direction of the effect remained the same, where children with two or more GP-recorded addresses were less likely to receive their MMR1 vaccination by 24 months of age.

EHRs are a source of routinely-collected administrative data, and do not provide information about the motivation for moving home. Linkage to other data sources, such as the Census, could provide additional information, for example about property tenure, which could aid interpretation of residential mobility. This was not in the scope of this study.

Whilst our study has focused on receipt of the MMR1 vaccination by 24 months of age, it is important to recognise that some children may receive a delayed vaccination after 24 months of age, and a second dose, now due at approximately 18 months of age, is essential for full protection [2]. Additional research investigating receipt of delayed vaccinations and the second MMR vaccination by age five years would further our understanding and improve identification of children with increased measles susceptibility.

### Comparison with existing literature

Our finding supports research in Canada which found the likelihood of being incompletely immunised by age seven was greater in children who had moved residence two times or more, compared to those who had moved one time or less. It is hypothesised that increased residential mobility may inhibit the development of long-term relationships with GPs and other healthcare workers, as well as bringing additional logistical barriers to vaccination which may arise due the chaos and competing priorities of residential instability [11].

Our estimates of residential mobility are considerably lower than those estimated from a nationally representative sample of children participating in the Millennium Cohort study [12, 26], although this difference is likely explained by the use of a longer follow-up period (up to age five years) in these studies.

Our findings are not consistent with evidence from the same national UK cohort, which found no difference in parentally-reported MMR1 immunisation status at three years of age among children who moved between birth and three years of age compared to those who did not move [11]. Similarly, in Wales it was reported that moving home frequently did not increase the odds of not being immunised when compared to not moving home [10]. As well as looking at coverage, they also reported that residential mobility did not impact on the timeliness of the receipt of vaccinations by 13 months of age. It is possible that we identify a different association between residential mobility and MMR1 vaccination due to differences in our study populations and definitions of timeliness. In NEL, the population is highly mobile, ethnically diverse and disadvantaged, with historically low uptake of MMR1 vaccination.

More broadly, our findings support other studies exploring the relationship between residential mobility and adverse health outcomes. For example, a study in New Zealand reported higher risk of potentially avoidable hospitalisations among children with higher residential mobility in the first 24 months of life [7], and in Wales, researchers found increased incidence of hospitalisations for infectious diseases and asthma among those with two or more moves [8].

### Implications for research, policy and practice

Our research has contributed to a growing body of evidence around the home environment and subsequent health behaviours and outcomes. Managing the health care needs of children in mobile population groups is a challenge, especially when moving between different healthcare providers. It is possible that residential mobility leads to a lack of continuity in primary care, and is a potentially contributing factor to incomplete immunisation status [27]. However, not all address changes will result in a change of GP and we did not look at change in registered GP with residential mobility so cannot make this inference. It is possible that residential mobility, and the consequential administrative process of changing GP, may result in delayed vaccination. We were unable to explore change in registered GP with residential mobility.

As well as the direct impact of residential mobility, it is also likely that residential mobility is associated with unobserved characteristics which lead to lower vaccination rates. Residential mobility may arise as a consequence of opposing socioeconomic circumstances. For some, residential mobility may indicate financial insecurity as families navigate short-term rental accommodation and perhaps more precarious housing, yet for others, residential mobility may signify improved financial security as families move towards home ownership [26]. It remains that residential mobility is driven by a range of factors including changes in employment, partnerships, family size and composition, and housing tenure [28]. Whatever the motivation for moving home, we estimate a population attributable risk percentage of 18.4% (supplementary file 3 table 11). This means that if no children experienced a change in address, 18.4% more children would be expected to receive a MMR1 vaccination by 24 months of age, assuming a causal association.

Measles vaccination may currently receive less priority in a health care system facing multiple challenges and clinical priorities [29]. The need for targeted public health interventions around routine childhood vaccinations has been recognised internationally [30, 31]. In England, a 2023-2024 national catch-up campaign by the UK Health Security Agency vaccinated more than 50,000 children aged 15 months to five years against MMR1 [32]. There is strong evidence to support the effectiveness of primary care led quality improvement programmes to improve vaccine uptake [33]. National measures to tackle these inequalities include NHS England’s Quality and Outcomes Frameworks to incentivise timely routine childhood vaccinations in primary care [34].

While there is technical guidance for the registration of new patients [35], good practice guidance on what happens once the patient is registered has not been developed. Consequently, the process for documenting and managing the registration process, and any clinical review of new patients, varies from GP to GP. Where residential mobility results in a change of GP practice, at the very least, a desktop patient review should be conducted to check vaccination status. Where GPs have the capacity to offer in-person new patient health checks, a focus on vaccinations would identify children overdue for any routine primary vaccinations. In practices where new patient health checks are not offered, opportunistic conversations about vaccinations with caregivers during children’s consultations for other issues might remind and/or encourage caregivers to make vaccination appointments. It is however important to recognise that opportunistic conversations should only be instigated by staff with appropriate training and should align with the GP practice strategy. It’s possible that published good practice guidance on the GP registration process could begin to standardise this process to ensure that the vaccination status of all children is checked as early as possible after registration, to improve the timeliness of vaccination receipt.

As well as new registration checks, an emphasis on making appointments as accessible as possible could facilitate vaccination uptake. We found that residential mobility in NEL is more common in the most deprived areas, and among households with a single adult or many children. These families may face several barriers to accessing vaccination appointments, including juggling employment and childcare, with limited time to make and attend appointments for multiple children. Increasing the availability of flexible, mobile services could facilitate vaccination uptake among those most likely to experience residential mobility. A pilot was launched in January 2026 to trial provision of vaccinations during routine health visitor visits - this model of care may be a step towards flexible, mobile services that are required by families facing barriers to accessing routine vaccination appointments [36]. Qualitative research focusing on understanding the implications of residential mobility for children and their families could highlight how services might be adapted to better suit the needs of those experiencing residential mobility.

## Conclusion

MMR1 vaccination coverage in NEL is well below the 95% recommended to achieve herd immunity. This is particularly important given the recent rise in measles cases in London. Our study adds important new evidence about the impact of residential mobility on MMR1 vaccination coverage. This provides further evidence to prioritise targeting those at greatest risk, to achieve herd immunity and prevent measles outbreaks.

## Supplementary Files

Supplementary Files

## Data Availability

Access to primary care data is enabled by data sharing agreements between the Discovery Data Service and the data controllers. The Discovery Programme Board has approved data access for the Research EnAbled Learning (REAL) Child Health programme for research on the condition that it is not onwardly shared.

## Abbreviations

ASSIGN:	AddreSS MatchInG to Unique Property Reference Numbers
CI:	confidence interval
COVER:	Cover of Vaccination Evaluated Rapidly
COVID-19:	2019 coronavirus
EHRs:	electronic health records
GPs:	general practices
IDACI:	Income Deprivation Affecting Children Index
LSOA:	lower layer super output area
MMR:	measles, mumps, and rubella
MMR1:	first measles, mumps, and rubella
NEL:	north-east London
NHS:	National Health Service
OR:	odds ratio
RALFs:	residential anonymised linkage fields
RECORD:	Reporting of studies Conducted using Observational Routinely-collected health Data Statement
SNOMED:	Systematized Medical Nomenclature for Medicine
UK:	United Kingdom
UPRN:	unique property reference number

## References

[1] Guerra FM, Bolotin S, Lim G, Heffernan J, Deeks SL, Li Y, et al. The basic reproduction number (R0) of measles: a systematic review. The Lancet Infectious Diseases. 2017;17(12):e420-e8. 10.1016/S1473-3099(17)30307-9 28757186

[2] Public Health England. Measles: the green book, chapter 21. In: Public Health England, editor. The Green Book 2019.

[3] UK Health Security Agency. Cover of vaccination evaluated rapidly (COVER) programme 2022 to 2023: quarterly data 2023 [Available from: https://www.gov.uk/government/statistics/cover-of-vaccination-evaluated-rapidly-cover-programme-2022-to-2023-quarterly-data.

[4] UK Health Security Agency. Confirmed cases of measles in England by month, age, region and upper tier local authority: 2024 2024 [Available from: https://www.gov.uk/government/publications/measles-epidemiology-2023/confirmed-cases-of-measles-in-england-by-month-age-region-and-upper-tier-local-authority-2024.

[5] O’Donnell J, Kingsley M. The relationship between housing and children’s socio-emotional and behavioral development in Australia. Children and Youth Services Review. 2020;117:105290. 10.1016/j.childyouth.2020.105290

[6] Nathan K, Robertson O, Carr PA, Howden-Chapman P, Pierse N. Residential mobility and socioemotional and behavioural difficulties in a preschool population cohort of New Zealand children. Journal of Epidemiology and Community Health. 2019;73(10):947-53. 10.1136/jech-2019-212436 31315898

[7] Nathan K, Robertson O, Atatoa Carr P, Howden-Chapman P, Pierse N. Residential mobility and potentially avoidable hospitalisations in a population-based cohort of New Zealand children. J Epidemiol Community Health. 2022;76(6):606-12. 10.1136/jech-2021-218509 35292510

[8] Hutchings HA, Evans A, Barnes P, Demmler JC, Heaven M, Healy MA, et al. Residential Moving and Preventable Hospitalizations. Pediatrics. 2016;138(1). 10.1542/peds.2015-2836 27260695

[9] Hutchings HA, Evans A, Barnes P, Demmler J, Heaven M, Hyatt MA, et al. Do Children Who Move Home and School Frequently Have Poorer Educational Outcomes in Their Early Years at School? An Anonymised Cohort Study. PLOS ONE. 2013;8(8):e70601. 10.1371/journal.pone.0070601 23940601 PMC3734306

[10] Hutchings HA, Evans A, Barnes P, Healy MA, James-Ellison M, Lyons RA, et al. Does frequent residential mobility in early years affect the uptake and timeliness of routine immunisations? An anonymised cohort study. Vaccine. 2016;34(15):1773-7. 10.1016/j.vaccine.2016.02.049 26923454 PMC4820086

[11] Pearce A, Elliman D, Bedford H, Law C. Residential mobility and uptake of childhood immunisations: Findings from the UK Millennium Cohort Study. Vaccine. 2008;26(13):1675-80. 10.1016/j.vaccine.2008.01.031 18294744

[12] Gambaro L, Joshi H. Moving home in the early years: what happens to children in the UK? Longitudinal and Life Course Studies. 2016;7(3). 10.14301/llcs.v7i3.375

[13] Morris T, Manley D, Northstone K, Sabel CE. How do moving and other major life events impact mental health? A longitudinal analysis of UK children. Health & Place. 2017;46:257-66. 10.1016/j.healthplace.2017.06.004 28666235

[14] Tseliou F, Maguire A, Donnelly M, O’Reilly D. The impact of childhood residential mobility on mental health outcomes in adolescence and early adulthood: a record linkage study. Journal of Epidemiology and Community Health. 2016;70(3):278-85. 10.1136/jech-2015-206123 26475920

[15] Vidal S, Baxter J. Residential relocations and academic performance of Australian children: A longitudinal analysis. Longitudinal and Life Course Studies. 2018;9(2). 10.14301/llcs.v9i2.435

[16] Vanhoutte B, Wahrendorf M, Nazroo J. Duration, timing and order: How housing histories relate to later life wellbeing. Longitudinal and Life Course Studies. 2017;8(3). 10.14301/llcs.v8i3.445

[17] Dhungana M, Hoben M, O’Brien C, MacDonald SE. Immunization status of children at kindergarten entry in Alberta, Canada. Canadian Journal of Public Health. 2023;114(1):82-92. 10.17269/s41997-022-00663-3 35864307 PMC9849539

[18] NHS England. About information governance: National Health Service; [Available from: https://www.england.nhs.uk/ig/about/].

[19] Harper G, Stables D, Simon P, Ahmed Z, Smith K, Robson J, et al. Evaluation of the ASSIGN open-source deterministic address-matching algorithm for allocating unique property reference numbers to general practitioner-recorded patient addresses. Int J Popul Data Sci. 2021;6(1):1674. 10.23889/ijpds.v6i1.1674 34970633 PMC8678979

[20] Harper G, Firman N, Wilk M, Marszalek M, Simon P, Stables D, et al. Determining households from patient addresses and unique property reference numbers in general practitioner electronic health records. International Journal of Population Data Science. 2024;9(1). 10.23889/ijpds.v9i1.2379 PMC11626511 39654832

[21] Firman N, Wilk M, Marszalek M, Griffiths L, Harper G, Dezateux C. Is obesity more likely among children sharing a household with an older child with obesity? Cross-sectional study of linked National Child Measurement Programme data and electronic health records. BMJ Paediatrics Open. 2024;8(1):e002533. https://doi.org/ 10.1136/bmjpo-2024-002533 38599801 10.1136/bmjpo-2024-002533 PMC11015308

[22] NHS. Ethnic category code: NHS Digital; [Available from: https://www.datadictionary.nhs.uk/data_elements/ethnic_category.html?hl=ethnic%2Ccategory].

[23] Harper G, Mayhew L. Using Administrative Data to Count and Classify Households with Local Applications. Applied Spatial Analysis and Policy. 2015;9(4):433-62. 10.1007/s12061-015-9162-2

[24] Ministry of Housing Communities & Local Government. The English Indices of Deprivation 2019 - Frequently Asked Questions (FAQs) 2016 [Available from: https://assets.publishing.service.gov.uk/government/uploads/system/uploads/attachment_data/file/853811/IoD2019_FAQ_v4.pdf.

[25] NHS Digital. Read Codes: NHS Digital; [Available from: https://digital.nhs.uk/services/terminology-and-classifications/read-codes].

[26] Gambaro L, Joshi H, Lupton R. Moving to a better place? Residential mobility among families with young children in the Millennium Cohort Study. Population, Space and Place. 2017;23(8):e2072. 10.1002/psp.2072

[27] Bailey GA, Lee A, Bedford H, Perry M, Holland S, Walton S, et al. Immunisation status of children receiving care and support in Wales: a national data linkage study. Frontiers in Public Health. 2023;11. 10.3389/fpubh.2023.1231264 PMC10423803 37583884

[28] Beck B, Buttaro A, Lennon MC. Home moves and child wellbeing in the first five years of life in the United States. Longitudinal and Life Course Studies. 2016;7(3). 10.14301/llcs.v7i3.374

[29] Bedford H, Donovan H. We need to increase MMR vaccine uptake urgently. BMJ. 2022;376:o.818 10.1136/bmj.o818 35354576

[30] MacDonald NE, Comeau JL, Dube E, Bucci LM. COVID-19 and missed routine immunizations: designing for effective catch-up in Canada. Can J Public Health. 2020;111(4):469-72. 10.17269/s41997-020-00385-4 32761546 PMC7408971

[31] World Health Organization. Guiding principles for immunization activities during the COVID-19 pandemic: interim guidance, 26 March 2020 Geneva: World Health Organization; 2020 [updated 2020. Available from: https://apps.who.int/iris/handle/10665/331590].

[32] UK Health Security Agency. Evaluating the impact of national and regional measles catch-up activity on MMR vaccine coverage in England, 2023 to 2024. UK Health Security Agency; 2024.

[33] Cockman P, Dawson L, Mathur R, Hull S. Improving MMR vaccination rates: herd immunity is a realistic goal. BMJ. 2011;343:d5703. 10.1136/bmj.d5703 21971162

[34] British Medical Association, NHS England. Update to the GP contract agreement 2020/21 - 2023/24: Primary Care Strategy and NHS Contracts Group; [Available from: https://www.england.nhs.uk/wp-content/uploads/2020/03/update-to-the-gp-contract-agreement-v2-updated.pdf].

[35] NHS Digital. GP registration digital guide: NHS Digital; 2024 [Available from: https://digital.nhs.uk/services/guides/gp-registration].

[36] Institute of Health Visiting. Government commits to prioritising child health – including childhood vaccination pilot 2026 [Available from: https://ihv.org.uk/news-and-views/news/government-commits-to-prioritising-child-health-including-childhood-vaccination-pilot/].

