# Supplementary material for: Residential mobility and receipt of measles, mumps and rubella vaccination: analysis of linked primary care electronic health records in a London region
Source: Int J Popul Data Sci. 2026 Mar 25;11(1):2963. doi: 10.23889/ijpds.v11i1.2963 (PMC13213897; doi:10.23889/ijpds.v11i1.2963)
Supplement: Supplementary Files [file ijpds-11-2963-s001.pdf]

Supplementary file 1 – Study protocol

Preliminary title

Residential mobility and receipt of measles, mumps and rubella vaccination: analysis of linked primary care electronic health records in a disadvantaged London region

Writing group

Nicola Firman, Laura North, Milena Marszalek, Marta Wilk, Ana Gutierrez, Rhodri Johnson, Carol Dezateux

Objective

To examine the association between residential mobility, assessed by the number of general practice (GP)-recorded addresses in the first two years of life, and receipt of MMR vaccination by age two years.

Study design

Retrospective longitudinal cohort study

Background

Measles is the most contagious disease, with a reproduction number between 12 and 18 [1]. Measles, mumps, and rubella (MMR) vaccination is the most effective public health measure to prevent measles infection, and while children are eligible for the first dose of the vaccine at 12 months in the United Kingdom (UK) [2], we are failing to vaccinate children on time. Coverage in the UK, that is the proportion of children receiving the vaccination between 12 and 24 months of age, is around 89% [3]. The north-east London (NEL) region has the lowest coverage in the UK at around 80% [3], well below the 95% recommended to achieve herd immunity. This low coverage is likely to have contributed to almost 2,563 laboratory confirmed cases of measles in England, the majority in London, in the first ten months of 2024 [4]. It has been hypothesised that children who move home frequently are less likely to receive vaccinations.

A child's home environment may affect their health and development, through the social and economic stability of the environment, the quality of the physical space, and the household members sharing and shaping use of the environment [5–15]. A growing body of literature suggests that residential mobility during childhood may be associated with adverse health and education outcomes throughout childhood, adolescence and into adulthood [16].

It has been shown that children who have moved home at least twice in the first year of life were more likely to attend hospital for ear, nose and throat infections, epilepsy, injuries, gastroenteritis, asthma, influenza, and dental conditions [8]. There is mixed evidence about the association between residential mobility and receipt of childhood vaccinations. No difference in the rates of coverage of childhood immunisations among children living in Wales and experiencing residential mobility, compared to those who did not. Similar findings have been reported in England, where researchers found children were less likely to have received the first MMR vaccination by three years of age, if they had moved home twice or more, compared to not moving, between the ages of nine months and three years [11]. Conversely, in Canada, the likelihood of being incompletely immunised by age seven was greater in children who had moved residence two times or more, compared to those who had moved one time or less [17].

Given these conflicting findings and the lack of studies focused in urban areas with high residential mobility, we examined associations between residential mobility in an ethnically diverse, disadvantaged population with low MMR vaccine uptake. We hypothesised that children experiencing residential mobility in the first 24 months would be less likely to receive the first MMR vaccination between 12 and 24 months of age, compared with those without residential mobility.

Target and observed population

The target population is all children living in north-east London (NEL) and eligible to receive their first MMR vaccination between 1st January 2015 and 30th October 2021. The observed population is all children registered with a NEL general practitioner (GP) on their second birthday between 1st January 2016 and 30th October 2021. These children would have been born between 1st January 2014 and 30th October 2019.

Data

Pseudonymised data will be provided from the NEL Discovery Data Service (DDS) which receives primary care electronic health record data on a daily basis from all general practices in NEL. Demographic and clinical data will be extracted for children ever registered with a NEL general practitioner, including children who may have died or left the area. Data extracts will include all clinical events up to 30th October 2021.

Unique property reference numbers

Linking people to place has become increasingly important in understanding the contexts of health inequalities. One method of doing this is by using Unique Property Reference Numbers (UPRNs) which are 12-digit unique identifiers for every addressable location in Great Britain [18]. UPRNs are now mandated in data collection across the public sector, acting as an address standardiser and a household identifier [19]. Every addressable location in Great Britain is assigned a UPRN by Ordnance Survey and local authorities. UPRNs identify a place of residence at a more granular level than lower super output area (LSOA) or postcode, identifying individual properties, for example houses or flats within a block or building shell.

The open source AddreSS MatchInG to Unique Property Reference Numbers (ASSIGN) algorithm has been developed by colleagues in the Clinical Effectiveness Group (CEG) and Endeavour Health Charity for use with National Health Service (NHS) recorded patient addresses. The algorithm provides

an accurate and validated method for assigning UPRNs, by matching GP-recorded addresses to those documented in AddressBase Premium (ABP), Ordnance Survey's Great Britain property gazetteer database.

Since UPRNs are a proxy address identifier, they are considered identifiable. However, their format enables pseudonymisation using encryption keys. Encrypted UPRNs are recorded as Residential Anonymised Linkage Fields (RALFs). RALFs are de-identified and are therefore usable in this research without disclosure of place of residence [20].

Discovery data service

The DDS brings together health-related data at the individual level, for a defined residential population for the purposes of population-based decision support to benefit health. The service contains the primary care records of people who have been registered with a NEL GP since 2014. The data are submitted by healthcare providers of primary, secondary and urgent care services and are updated in near real time. The DDS has collected data in NEL since 2014, and continues to receive coded data using nationally recognised Systematized Nomenclature of Medicine clinical terms (SNOMED CT), as well as system-specific codes relating to the software systems provider e.g. Egton Medical Information Systems (EMIS) and SystmOne.

In addition to clinical data, the ASSIGN algorithm has been deployed in the DDS database, identifying UPRNs for 98.6% of GP-recorded patient addresses [18]. UPRNs and their associated metadata are retained in DDS. This process is repeated in near real time as new patient addresses enter the DDS. UPRNs are pseudonymised into RALFs within DDS, using a study-specific encryption key. Data flow into NEL DDS began in 2014 and the database contains only address records that were current for a registration at that point in time and address changes since then.

Ethics

This study work analyses routinely acquired de-identified data. Access to primary care data is enabled by data sharing agreements between the DDS and NEL GP and Barts NHS Trust data controllers. The Discovery Programme Board has approved data access by the REAL Child Health programme.

Information governance

All data will be extracted and managed according to UK NHS information governance requirements [21].

Cohort definition

All children registered with a NEL GP on their second birthday between 1st January 2016 and 30th October 2021 will be eligible for inclusion.

Exclusion criteria

Children will be excluded from the cohort if they:

- Do not have a regular GP registration in NEL on their second birthday
- Do not have a residential RALF on their second birthday
- Have more than one regular GP registration on their second birthday
- Have unknown sex
- Are living without any other household members aged 0-100 years on their second birthday
- Are living with more than nine other people on their second birthday
- Are not living with any one aged 18-100 years on their second birthday

Variables of interest

Outcome variable

Table 1: Outcomes of interest

Variable	Description
MMR status	A binary variable indicting if the child has received their first MMR vaccination between 12 and 24 months of age. The SNOMED clinical code specification can be found in table 2.

Table 2: Systemized Nomenclature of Medicine (SNOMED) – Clinical Terms for first measles, mumps and rubella vaccination procedures

SNOMED concept ID	Code description
38598009	Administration of measles and mumps and rubella vaccine
432636005	Administration of measles and mumps and rubella and varicella virus vaccine
713404003	Measles, mumps and rubella vaccination given (situation)
871909005	Administration of first dose of measles and mumps and rubella and varicella virus vaccine
150971000119104	[V]Measles-mumps-rubella (MMR) vaccination
308081000000105	First MMR (measles mumps and rubella) vaccination
505001000000109	Measles mumps rubella catch-up vaccination
571591000119106	Administration of live attenuated measles mumps and rubella vaccine
1037251000000100	First MMR vaccination given by other healthcare provider
82314000	Rubella vaccination*
50583002	Mumps vaccination*
47435007	Measles vaccination*

Events recorded in the primary care electronic health record using another clinical coding system (e.g. Read v2 or EMIS local codes) have been mapped to relevant SNOMED codes within the Discovery Data Service. This ensures that searching the database using SNOMED codes captured all events regardless of the clinical coding system used. Codes for separate measles, mumps, and rubella vaccinations were included to account for historic use of separate component codes in general practices using Vision or SystmOne software, where vaccinations got recorded as separate components, even if they were administered as one vaccine.

Explanatory variable

Table 3: Explanatory variable of interest

Variable	Description
Residential mobility	A discrete count of unique RALFs between birth (or earliest NEL registration) and either second birthday or MMR vaccination date. Periods of no GP registration in NEL lasting more than 30 days will be counted as one additional RALF. An additional RALF will also be counted for children whose first NEL GP registration starts when they are aged >60 days old. Categorised into: 1, 2, 3 or more.<br>See figure 1.

Figure 1: Counting residential anonymised linkage fields

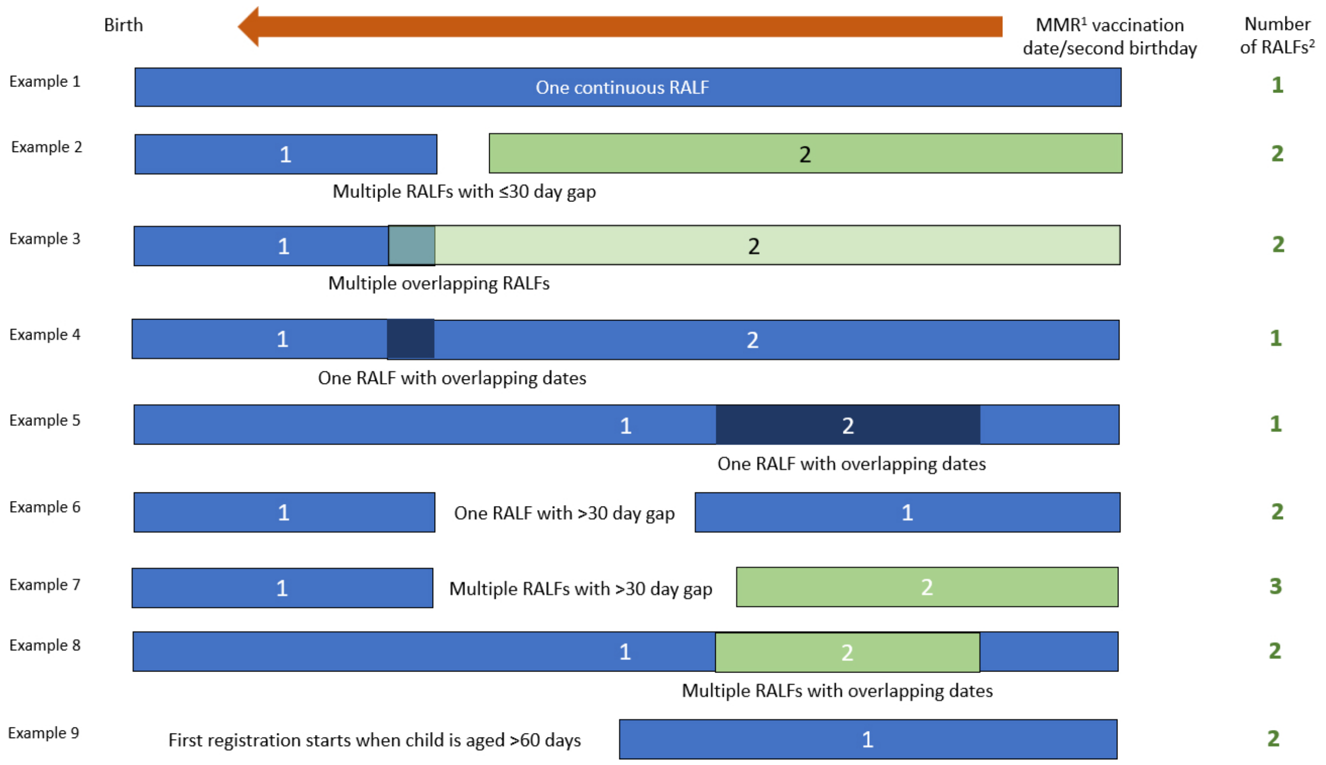

[1]Measles, mumps and rubella. [2]Residential anonymised linkage fields.

Covariates

Table 3: Covariates

Variable	Description
Sex	General Practice-recorded sex as coded in the cohort child's GP registration, either:<br>1. Male<br>2. Female
Ethnic background	General Practice-recorded ethnic background as coded in the cohort child's GP registration categorised into the NHS 16+1 groups:<br>1. White British<br>2. White Irish<br>3. Other White<br>4. Chinese<br>5. White & Asian<br>6. White & Black African<br>7. White & Black Caribbean<br>8. Other Asian<br>9. Other Mixed<br>10. Other<br>11. Bangladeshi<br>12. Indian<br>13. Pakistani<br>14. Black African<br>15. Black Caribbean<br>16. Other Black
Number of children in the household	A categorical count of the number of children (aged 0-17.9 years) resident in the household on the child's second birthday. Categorised into: 1, 2, 3, 4 or more.
Household composition	Household composition on the child's second birthday, derived by calculating age for each household member. A modified Harper and Mayhew [22] method for categorising household composition will be used, counting the number of household members in three age brackets: 0-17 years (children, inclusive), 18-64 years (working age adults, inclusive) and 65 or older (older adults). Household composition will be comprised of the following categories:<br>1. Family with child(ren) (one or more children with two or more working age adults and no older adults)<br>2. Single adult with child(ren) (one or more children with one working age adult and no older adults)<br>3. Three generation (at least one child, at least one working age adult and at least one older adult) or skipped generation (at least one child and one older adult but no working age adults)<br>4. Other
Income Deprivation Affecting Children Index (IDACI)	The 2019 Income Deprivation Affecting Children Index quintile associated with the lower layer super output area (LSOA) where the property resided at on second birthday is located will be linked to each child's record. IDACI decile will be linked based on 2011 LSOA as recorded in the cohort child's GP record and concatenated into quintiles.
Local authority	Local authority associated with the cohort child's home address on their second birthday:<br>1. Barking & Dagenham<br>2. City & Hackney<br>3. Havering<br>4. Newham<br>5. Redbridge<br>6. Tower Hamlets<br>7. Waltham Forest

Statistical analyses

Analyses will be performed using Stata/MP 17 (StataCorp LP):

1. Summary statistics to describe the demographic characteristics of children in the study sample, as well as the proportion by MMR vaccination status and residential mobility.
2. Descriptive statistics to illustrate the proportion (and 95% confidence intervals) of children with and without MMR vaccination, by residential mobility and other covariates.
3. Descriptive statistics to illustrate the proportion (and 95% confidence intervals) of children experiencing residential mobility, by covariates.
4. Binary logistic regression to estimate univariable odds ratios (and 95% confidence intervals) for MMR vaccination by residential mobility.
5. Binary logistic regression to estimate multivariable odds ratios (and 95% confidence intervals) for MMR vaccination by residential mobility, using a stepwise approach introducing individual-, household-, and area-level covariates.

Sensitivity analyses

Sensitivity analyses will be conducted and reported in the results section. Tables and figures will be included in the supplementary material.

1. Repeat all analyses without adding RALFs for children who were aged greater than 60 days old when they first registered with a NEL GP or for each period of greater than 30 days when a child was not registered with a NEL GP.
2. Repeat all analyses on a cohort of children registered with a NEL GP on their second birthday between 1st January 2016 and 20th March 2020. The receipt of MMR vaccination among these children is expected to be unaffected by the 2019 Coronavirus (COVID-19) pandemic.

Manuscript preparation

The STrengthening the Reporting of OBservational studies in Epidemiology (STROBE) guidelines will be followed.

References

1. Guerra FM, Bolotin S, Lim G, Heffernan J, Deeks SL, Li Y, et al. The basic reproduction number (R0) of measles: a systematic review. The Lancet Infectious Diseases. 2017;17(12):e420-e8.
2. Public Health England. Measles: the green book, chapter 21. In: Public Health England, editor. The Green Book2019.
3. UK Health Security Agency. Cover of vaccination evaluated rapidly (COVER) programme 2022 to 2023: quarterly data 2023 [Available from: https://www.gov.uk/government/statistics/cover-of-vaccination-evaluated-rapidly-cover-programme-2022-to-2023-quarterly-data.
4. UK Health Security Agency. Confirmed cases of measles in England by month, age, region and upper tier local authority: 2024 2024 [Available from: https://www.gov.uk/government/publications/measles-epidemiology-2023/confirmed-cases-of-measles-in-england-by-month-age-region-and-upper-tier-local-authority-2024.
5. O'Donnell J, Kingsley M. The relationship between housing and children's socio-emotional and behavioral development in Australia. Children and Youth Services Review. 2020;117:105290.
6. Nathan K, Robertson O, Carr PA, Howden-Chapman P, Pierse N. Residential mobility and socioemotional and behavioural difficulties in a preschool population cohort of New Zealand children. Journal of Epidemiology and Community Health. 2019;73(10):947-53.
7. Nathan K, Robertson O, Atatoa Carr P, Howden-Chapman P, Pierse N. Residential mobility and potentially avoidable hospitalisations in a population-based cohort of New Zealand children. J Epidemiol Community Health. 2022;76(6):606-12.
8. Hutchings HA, Evans A, Barnes P, Demmler JC, Heaven M, Healy MA, et al. Residential Moving and Preventable Hospitalizations. Pediatrics. 2016;138(1).
9. Hutchings HA, Evans A, Barnes P, Demmler J, Heaven M, Hyatt MA, et al. Do Children Who Move Home and School Frequently Have Poorer Educational Outcomes in Their Early Years at School? An Anonymised Cohort Study. PLOS ONE. 2013;8(8):e70601.
10. Hutchings HA, Evans A, Barnes P, Healy MA, James-Ellison M, Lyons RA, et al. Does frequent residential mobility in early years affect the uptake and timeliness of routine immunisations? An anonymised cohort study. Vaccine. 2016;34(15):1773-7.
11. Pearce A, Elliman D, Bedford H, Law C. Residential mobility and uptake of childhood immunisations: Findings from the UK Millennium Cohort Study. Vaccine. 2008;26(13):1675-80.
12. Gambaro L, Joshi H. Moving home in the early years: what happens to children in the UK? Longitudinal and Life Course Studies. 2016;7(3).
13. Morris T, Manley D, Northstone K, Sabel CE. How do moving and other major life events impact mental health? A longitudinal analysis of UK children. Health & Place. 2017;46:257-66.

14. Tseliou F, Maguire A, Donnelly M, O'Reilly D. The impact of childhood residential mobility on mental health outcomes in adolescence and early adulthood: a record linkage study. Journal of Epidemiology and Community Health. 2016;70(3):278-85.

15. Vidal S, Baxter J. Residential relocations and academic performance of Australian children: A longitudinal analysis. Longitudinal and Life Course Studies. 2018;9(2).

16. Vanhoutte B, Wahrendorf M, Nazroo J. Duration, timing and order: How housing histories relate to later life wellbeing. Longitudinal and Life Course Studies. 2017;8(3).

17. Dhungana M, Hoben M, O'Brien C, MacDonald SE. Immunization status of children at kindergarten entry in Alberta, Canada. Canadian Journal of Public Health. 2023;114(1):82-92.

18. Harper G, Stables D, Simon P, Ahmed Z, Smith K, Robson J, et al. Evaluation of the ASSIGN open-source deterministic address-matching algorithm for allocating unique property reference numbers to general practitioner-recorded patient addresses. Int J Popul Data Sci. 2021;6(1):1674.

19. Ordnance Survey. The Public Sector Geospatial Agreement: Providing the public sector access to our world-leading geospatial data: Ordnance Survey; [Available from: https://www.ordnancesurvey.co.uk/customers/public-sector/public-sector-geospatial-agreement.

20. Rodgers SE, Lyons RA, Dsilva R, Jones KH, Brooks CJ, Ford DV, et al. Residential Anonymous Linking Fields (RALFs): a novel information infrastructure to study the interaction between the environment and individuals' health. Journal of Public Health. 2009;31(4):582-8.

21. NHS England. About information governance: National Health Service; [Available from: https://www.england.nhs.uk/ig/about/.

22. Harper G, Mayhew L. Using Administrative Data to Count and Classify Households with Local Applications. Applied Spatial Analysis and Policy. 2015;9(4):433-62.

Supplementary file 2 – The RECORD statement - checklist of items, extended from the STROBE statement, that should be reported in observational studies using routinely collected health data.

	Item No.	STROBE items	Location in manuscript where items are reported	RECORD items	Location in manuscript where items are reported
**Title and abstract**					
	1	(a) Indicate the study's design with a commonly used term in the title or the abstract (b) Provide in the abstract an informative and balanced summary of what was done and what was found	Abstract (page 2)	RECORD 1.1: The type of data used should be specified in the title or abstract. When possible, the name of the databases used should be included.<br>RECORD 1.2: If applicable, the geographic region and timeframe within which the study took place should be reported in the title or abstract.<br>RECORD 1.3: If linkage between databases was conducted for the study, this should be clearly stated in the title or abstract.	Title & abstract (pages 1 & 2)<br>Title & abstract (pages 1 & 2)<br>Title & abstract (pages 1 & 2)
**Introduction**					
Background rationale	2	Explain the scientific background and rationale for the investigation being reported	Introduction (page 3)		
Objectives	3	State specific objectives, including any prespecified hypotheses	Introduction (page 3)		
**Methods**					
Study Design	4	Present key elements of study design early in the paper	Methods (page 4)		
Setting	5	Describe the setting, locations, and relevant dates, including periods of recruitment, exposure, follow-up, and data collection	Methods (page 4)		
Participants	6	*(a) Cohort study* - Give the eligibility criteria, and the sources and methods of selection of participants. Describe methods of follow-up<br>*Case-control study* - Give the eligibility criteria, and the sources and methods of case ascertainment and control selection. Give the rationale for the choice of cases and controls<br>*Cross-sectional study* - Give the eligibility criteria, and the sources and methods of selection of participants<br>*(b) Cohort study* - For matched studies, give matching criteria and number of exposed and unexposed<br>*Case-control study* - For matched studies, give matching criteria and the number of controls per case	Methods (pages 4-6)<br>n/a	RECORD 6.1: The methods of study population selection (such as codes or algorithms used to identify subjects) should be listed in detail. If this is not possible, an explanation should be provided.<br>RECORD 6.2: Any validation studies of the codes or algorithms used to select the population should be referenced. If validation was conducted for this study and not published elsewhere, detailed methods and results should be provided.<br>RECORD 6.3: If the study involved linkage of databases, consider use of a flow diagram or other graphical display to demonstrate the data linkage process, including the number of individuals with linked data at each stage.	Methods (pages 4-6)<br>n/a<br>Figure 1
Variables	7	Clearly define all outcomes, exposures, predictors, potential confounders, and effect modifiers. Give diagnostic criteria, if applicable.	Methods (pages 6-7)	RECORD 7.1: A complete list of codes and algorithms used to classify exposures, outcomes, confounders, and effect modifiers should be provided. If these cannot be reported, an explanation should be provided.	Methods (pages 6-7) & supplementary file 3 table 2
Data sources/ measurement	8	For each variable of interest, give sources of data and details of methods of assessment (measurement).<br>Describe comparability of assessment methods if there is more than one group	Methods (pages 6-8)		
Bias	9	Describe any efforts to address potential sources of bias	Methods (page 8)		

Continued

	Item No.	STROBE items	Location in manuscript where items are reported	RECORD items	Location in manuscript where items are reported
Study size	10	Explain how the study size was arrived at	Methods (pages 4-6) & figure 1		
Quantitative variables	11	Explain how quantitative variables were handled in the analyses. If applicable, describe which groupings were chosen, and why	Methods (pages 6-7)		
Statistical methods	12	(a) Describe all statistical methods, including those used to control for confounding<br>(b) Describe any methods used to examine subgroups and interactions<br>(c) Explain how missing data were addressed<br>(d) *Cohort study* - If applicable, explain how loss to follow-up was addressed<br>*Case-control study* - If applicable, explain how matching of cases and controls was addressed<br>*Cross-sectional study* - If applicable, describe analytical methods taking account of sampling strategy<br>(e) Describe any sensitivity analyses	Methods (page 7)<br>Methods (page 7)<br>Methods (pages 4-6) & supplementary file 1 table 1<br>Methods (page 7)		
Data access and cleaning methods		..		RECORD 12.1: Authors should describe the extent to which the investigators had access to the database population used to create the study population.<br>RECORD 12.2: Authors should provide information on the data cleaning methods used in the study.	Methods (page 4)<br>Methods (page 4)
Linkage		..		RECORD 12.3: State whether the study included person-level, institutional-level, or other data linkage across two or more databases. The methods of linkage and methods of linkage quality evaluation should be provided.	Methods (page 4)
**Results**					
Participants	13	(a) Report the numbers of individuals at each stage of the study (*e.g.*, numbers potentially eligible, examined for eligibility, confirmed eligible, included in the study, completing follow-up, and analysed)<br>(b) Give reasons for non-participation at each stage.<br>(c) Consider use of a flow diagram	Results (page 8) & figure 1	RECORD 13.1: Describe in detail the selection of the persons included in the study (*i.e.*, study population selection) including filtering based on data quality, data availability and linkage. The selection of included persons can be described in the text and/or by means of the study flow diagram.	Methods (page 4), results (page 8) & figure 1
Descriptive data	14	(a) Give characteristics of study participants (*e.g.*, demographic, clinical, social) and information on exposures and potential confounders<br>(b) Indicate the number of participants with missing data for each variable of interest<br>(c) *Cohort study* - summarise follow-up time (*e.g.*, average and total amount)	Results (page 8) & table 1		
Outcome data	15	*Cohort study* - Report numbers of outcome events or summary measures over time<br>*Case-control study* - Report numbers in each exposure category, or summary measures of exposure<br>*Cross-sectional study* - Report numbers of outcome events or summary measures	Results (page 8) & table 1		

Continued

	Item No.	STROBE items	Location in manuscript where items are reported	RECORD items	Location in manuscript where items are reported
Main results	16	(a) Give unadjusted estimates and, if applicable, confounder-adjusted estimates and their precision (e.g., 95% confidence interval). Make clear which confounders were adjusted for and why they were included<br>(b) Report category boundaries when continuous variables were categorised<br>(c) If relevant, consider translating estimates of relative risk into absolute risk for a meaningful time period	Results (page 12) & figure 3		
Other analyses	17	Report other analyses done—e.g., analyses of subgroups and interactions, and sensitivity analyses	Results (pages 13-14) & figure 4		
**Discussion**					
Key results	18	Summarise key results with reference to study objectives	Discussion (pages 14-15)		
Limitations	19	Discuss limitations of the study, taking into account sources of potential bias or imprecision. Discuss both direction and magnitude of any potential bias	Discussion (pages 15-16)	RECORD 19.1: Discuss the implications of using data that were not created or collected to answer the specific research question(s). Include discussion of misclassification bias, unmeasured confounding, missing data, and changing eligibility over time, as they pertain to the study being reported.	Discussion (pages 15-16)
Interpretation	20	Give a cautious overall interpretation of results considering objectives, limitations, multiplicity of analyses, results from similar studies, and other relevant evidence	Discussion (pages 16-18)		
Generalisability	21	Discuss the generalisability (external validity) of the study results	Discussion (pages 16-17)		
**Other Information**					
Funding	22	Give the source of funding and the role of the funders for the present study and, if applicable, for the original study on which the present article is based	Funding (page 19)		
Accessibility of protocol, raw data, and programming code		..		RECORD 22.1: Authors should provide information on how to access any supplemental information such as the study protocol, raw data, or programming code.	Supplementary file 3 & data availability statement (page 19)

*Reference: Benchimol EI, Smeeth L, Guttmann A, Harron K, Moher D, Petersen I, Sørensen HT, von Elm E, Langan SM, the RECORD Working Committee. The REporting of studies Conducted using Observational Routinely-collected health Data (RECORD) Statement. *PLoS Medicine* 2015; in press.

*Checklist is protected under Creative Commons Attribution (CC BY) licence.

Supplementary file 3 – Additional tables and figures

Table 1: Sample characteristics of those excluded from the study sample (column percentages)

	Included in the study sample (n=150,949)			Excluded from the study sample (n=16,841)		
	**n**	**%**	**95% CI[1]**	**n**	**%**	**95% CI[1]**
**MMR[2] status**						
No MMR between 12 and 24 months	22991	15.2	15.1,15.4	2788	16.6	16.0,17.1
MMR between 12 and 24 months	127958	84.8	84.6,84.9	14053	83.4	82.9,84.0
**Number of GP-recorded addresses[3]**						
1	117156	77.6	77.4,77.8	8001	47.5	46.8,48.3
2	31769	21.0	20.8,21.3	1651	9.8	9.4,10.3
3 or more	2024	1.4	1.3,1.4	78	0.5	0.4,0.6
0 or unable to determine	0			7111	42.2	41.5,43.0
**Sex**						
Male	77036	51.0	50.8,51.3	8652	51.4	50.6,52.1
Female	73913	49.0	48.7,49.2	8188	48.6	47.9,49.4
Unknown	0			1	0.0	0.0,0.0
**Ethnic background**						
White British	27492	18.2	18.0,18.4	2578	15.3	14.8,15.9
White Irish	324	0.2	0.2,0.2	35	0.2	0.2,0.3
Other White	18617	12.3	12.2,12.5	2240	13.3	12.8,13.8
Chinese	936	0.6	0.6,0.7	91	0.5	0.4,0.7
White & Asian	1533	1.0	1.0,1.1	114	0.7	0.6,0.8
White & Black African	1142	0.8	0.7,0.8	119	0.7	0.6,0.8
White & Black Caribbean	1157	0.8	0.7,0.8	122	0.7	0.6,0.8
Other Asian	4497	3.0	2.9,3.1	438	2.6	2.4,2.9
Other Mixed	3206	2.1	2.1,2.2	363	2.2	1.9,2.4
Other	6442	4.3	4.2,4.4	1144	6.8	6.4,7.2
Bangladeshi	14350	9.5	9.3,9.6	1600	9.5	9.1,10.0
Indian	8062	5.3	5.2,5.5	807	4.8	4.5,5.1
Pakistani	9878	6.5	6.4,6.7	1056	6.3	5.9,6.6
Black African	7130	4.7	4.6,4.8	856	5.1	4.8,5.4
Black Caribbean	1421	0.9	0.9,1.0	210	1.2	1.1,1.4
Other Black	3800	2.5	2.4,2.6	546	3.2	3.0,3.5
Missing	40962	27.1	26.9,27.4	4522	26.9	26.2,27.5
**IDACI quintile[6]**						
1 - most deprived	61779	40.9	40.7,41.2	7632	45.3	44.6,46.1
2	56157	37.2	37.0,37.4	6509	38.6	38.0,39.4
3	21491	14.2	14.1,14.4	2001	11.9	11.4,12.4
4	8300	5.5	5.4,5.6	554	3.3	3.0,3.6
5 - least deprived	3191	2.1	2.0,2.2	137	0.8	0.7,1.0
Missing	31	0.0	0.0,0.0	8	0.0	
**Local authority**						
Barking	19376	12.8	12.7,13.0	636	3.8	3.5,4.1
City & Hackney	19589	13.0	12.8,13.1	4521	26.8	26.2,27.5
Havering	18264	12.1	12.0,12.3	820	4.9	4.6,5.2
Newham	26315	17.4	17.2,17.6	5572	33.1	32.4,33.8
Redbridge	24162	16.0	15.8,16.2	1303	7.7	7.3,8.2
Tower Hamlets	21029	13.9	13.7,14.1	2129	12.6	12.1,13.2
Waltham Forest	22214	14.7	14.5,14.9	1860	11.0	10.6,11.5

[1]Confidence interval. [2]Measles, mumps and rubella vaccination status. [3]General practice. [4]Number of people aged 0-17.9 years sharing the same address on the date of the child's second birthday. [5]Household composition defined on the date of the child's second birthday. [6]2019 Income Deprivation Affecting Children Index quintile.

Table 2: Systemized Nomenclature of Medicine (SNOMED) – Clinical Terms for first measles, mumps and rubella vaccination procedures

SNOMED concept ID	Code description
38598009	Administration of measles and mumps and rubella vaccine
432636005	Administration of measles and mumps and rubella and varicella virus vaccine
713404003	Measles, mumps and rubella vaccination given (situation)
871909005	Administration of first dose of measles and mumps and rubella and varicella virus vaccine
150971000119104	[V]Measles-mumps-rubella (MMR) vaccination
308081000000105	First MMR (measles mumps and rubella) vaccination
505001000000109	Measles mumps rubella catch-up vaccination
571591000119106	Administration of live attenuated measles mumps and rubella vaccine
1037251000000100	First MMR vaccination given by other healthcare provider
82314000	Rubella vaccination*
50583002	Mumps vaccination*
47435007	Measles vaccination*

Events recorded in the primary care electronic health record using another clinical coding system (e.g. Read v2 or EMIS local codes) have been mapped to relevant SNOMED codes within the Discovery Data Service. This ensures that searching the database using SNOMED codes captured all events regardless of the clinical coding system used. *Codes for separate measles, mumps, and rubella vaccinations were included to account for historic use of separate component codes in general practices using Vision or SystmOne software, where vaccinations were recorded as separate components, even if they were administered as one vaccine.

Table 3: Distribution of changes in general practice-recorded addresses

	Number of GP-recorded addresses[1]		
	n	%	95% CI[1]
1	108569	80.3	80.1,80.5
2	24950	18.5	18.2,18.7
3	1550	1.1	1.1,1.2
4	140	0.1	0.1,0.1
5	12	0.01	0.0,0.01

[1]General practice.

Table 4: Binary logistic regression models estimating odds ratios for measles, mumps and rubella vaccination by 24 months of age

	Univariable		Number of GP-recorded addresses + individual-level characteristics		Number of GP-recorded addresses + individual- and household-level characteristics		Number of GP-recorded addresses + individual-, household-, and area-level characteristics	
	OR[1]	95% CI[2]	OR[1]	95% CI[2]	OR[1]	95% CI[2]	OR[1]	95% CI[2]
**Number of GP-recorded addresses[3]**								
1 (reference)	1		1		1		1	
2	0.44	0.43,0.45	0.47	0.45,0.48	0.47	0.45,0.49	0.46	0.44,0.48
3 or more	0.31	0.28,0.34	0.33	0.29,0.36	0.33	0.30,0.37	0.32	0.29,0.36
**Sex**								
Male (reference)	1		1		1		1	
Female	1.03	1.00,1.06	1.03	1.00,1.07	1.04	1.00,1.07	1.04	1.00,1.07
**Ethnic background**								
White British (reference)	1		1		1		1	
White Irish	0.37	0.28,0.48	0.39	0.30,0.51	0.41	0.31,0.53	0.44	0.34,0.57
Other White	0.44	0.42,0.46	0.47	0.44,0.49	0.47	0.44,0.49	0.50	0.47,0.53
Chinese	1.36	1.07,1.73	1.44	1.13,1.84	1.35	1.06,1.72	1.37	1.08,1.75
White & Asian	0.95	0.81,1.13	0.97	0.82,1.15	0.95	0.80,1.12	1.00	0.85,1.19
White & Black African	0.69	0.58,0.81	0.73	0.61,0.86	0.77	0.65,0.92	0.82	0.69,0.97
White & Black Caribbean	0.46	0.40,0.53	0.48	0.41,0.56	0.50	0.43,0.58	0.53	0.45,0.61
Other Asian	0.98	0.89,1.09	0.99	0.89,1.10	1.07	0.96,1.18	1.14	1.02,1.27
Other Mixed	0.61	0.55,0.68	0.64	0.58,0.71	0.64	0.58,0.72	0.70	0.63,0.77
Other	0.31	0.29,0.33	0.32	0.30,0.35	0.39	0.36,0.41	0.49	0.45,0.52
Bangladeshi	1.20	1.12,1.29	1.22	1.14,1.31	1.38	1.29,1.49	1.33	1.23,1.44
Indian	0.90	0.83,0.97	0.96	0.89,1.04	0.97	0.90,1.05	1.08	0.99,1.17
Pakistani	0.88	0.82,0.95	0.90	0.84,0.97	1.06	0.99,1.15	1.18	1.09,1.27
Black African	0.63	0.59,0.68	0.66	0.61,0.71	0.77	0.71,0.83	0.83	0.77,0.90
Black Caribbean	0.31	0.27,0.35	0.32	0.28,0.36	0.34	0.30,0.39	0.39	0.34,0.44
Other Black	0.44	0.40,0.48	0.45	0.42,0.50	0.52	0.48,0.57	0.57	0.52,0.62
**Number of children in the household[4]**								
1 (reference)	1				1		1	
2	0.88	0.84,0.91			0.82	0.79,0.86	0.82	0.78,0.86
3	0.72	0.69,0.75			0.66	0.63,0.69	0.66	0.63,0.70
4 or more	0.47	0.45,0.48			0.44	0.42,0.46	0.46	0.44,0.49
**Household composition[5]**								
Adults with child(ren) (reference)	1				1		1	
Single adult with child(ren)	0.83	0.80,0.86			0.81	0.77,0.84	0.81	0.77,0.85
Other	1.03	0.98,1.08			0.88	0.82,0.93	0.87	0.82,0.93
**IDACI quintile[6]**								
1 - most deprived (reference)	1						1	
2	0.88	0.85,0.91					0.92	0.88,0.96
3	1.04	0.99,1.08					1.08	1.02,1.15
4	1.03	0.97,1.10					1.15	1.05,1.25
5 - least deprived	2.04	1.79,2.33					1.67	1.42,1.97
**Local authority**								
Barking	0.82	0.78,0.86					0.91	0.85,0.97
City & Hackney	0.52	0.49,0.54					0.61	0.58,0.65
Havering	1.46	1.38,1.55					1.39	1.29,1.50
Newham (reference)	1						1	
Redbridge	0.85	0.81,0.89					0.71	0.67,0.76
Tower Hamlets	1.46	1.38,1.55					1.15	1.07,1.25
Waltham Forest	0.92	0.88,0.97					0.91	0.86,0.97

[1]Odds ratio. [2]Confidence interval. [3]General practice. [4]Number of people aged 0-17.9 years sharing the same address on the date of the child's second birthday. [5]Household composition defined on the date of the child's second birthday. [6]2019 Income Deprivation Affecting Children Index quintile.

Sensitivity analysis 1: Analyses without adding RALFs for children who were aged greater than 60 days old when they first registered with a NEL GP or for each period of greater than 30 days when a child was not registered with a NEL GP

Table 5: Residential mobility, assessed by the number of general practice-recorded addresses, without adjustment for unregistered periods

	All children		
	**n**	**%**	**95% CI[1]**
**Number of GP-recorded addresses[2]**			
1	142562	94.4	94.3,94.6
2	8124	5.4	5.2,5.5
3 or more	263	0.2	0.2,0.2

[1]Confidence interval. [2]General practice.

Table 6: Residential mobility, assessed by number of general practice-recorded addresses, by first measles, mumps and rubella (MMR) vaccination status

	No MMR between 12 and 24 months			MMR between 12 and 24 months		
	**n**	**%**	**95% CI[1]**	**n**	**%**	**95% CI[1]**
**All**	22991	15.2	15.1,15.4	127958	84.8	84.6,84.9
**Number of GP-recorded addresses[2]**						
1	21406	15.0	14.8,15.2	121156	85.0	84.8,85.2
2	1518	18.7	17.9,19.5	6606	81.3	80.5,82.1
3 or more	67	25.5	20.6,31.1	196	74.5	68.9,79.4

[1]Confidence interval. [2]General practice.

Table 7: Binary logistic regression models estimating odds ratios for measles, mumps and rubella vaccination by 24 months of age

	Univariable		Number of GP-recorded addresses + individual-level characteristics		Number of GP-recorded addresses + individual- and household-level characteristics		Number of GP-recorded addresses + individual-, household-, and area-level characteristics	
	OR[1]	95% CI[2]	OR[1]	95% CI[2]	OR[1]	95% CI[2]	OR[1]	95% CI[2]
**Number of GP-recorded addresses[3]**								
1 (reference)	1		1		1		1	
2	0.77	0.73,0.81	0.77	0.72,0.82	0.75	0.71,0.81	0.74	0.69,0.79
3 or more	0.52	0.39,0.68	0.47	0.35,0.64	0.46	0.34,0.63	0.45	0.33,0.62
**Sex**								
Male (reference)	1		1		1		1	
Female	1.03	1.00,1.06	1.03	1.00,1.07	1.04	1.00,1.07	1.04	1.00,1.07
**Ethnic background**								
White British (reference)	1		1		1		1	
White Irish	0.37	0.28,0.48	0.37	0.28,0.48	0.38	0.29,0.49	0.41	0.31,0.53
Other White	0.44	0.42,0.46	0.44	0.42,0.46	0.44	0.42,0.47	0.47	0.45,0.50
Chinese	1.36	1.07,1.73	1.36	1.07,1.73	1.28	1.00,1.62	1.28	1.01,1.64
White & Asian	0.95	0.81,1.13	0.96	0.81,1.13	0.94	0.79,1.11	0.99	0.83,1.17
White & Black African	0.69	0.58,0.81	0.69	0.58,0.82	0.74	0.63,0.88	0.79	0.66,0.93
White & Black Caribbean	0.46	0.40,0.53	0.46	0.40,0.54	0.48	0.41,0.56	0.51	0.44,0.59
Other Asian	0.98	0.89,1.09	0.98	0.89,1.09	1.06	0.96,1.18	1.13	1.02,1.26
Other Mixed	0.61	0.55,0.68	0.62	0.56,0.68	0.62	0.56,0.69	0.67	0.61,0.75
Other	0.31	0.29,0.33	0.31	0.29,0.33	0.37	0.35,0.40	0.45	0.42,0.49
Bangladeshi	1.20	1.12,1.29	1.21	1.13,1.30	1.38	1.29,1.48	1.32	1.23,1.43
Indian	0.90	0.83,0.97	0.90	0.83,0.97	0.91	0.84,0.99	1.02	0.94,1.10
Pakistani	0.88	0.82,0.95	0.89	0.82,0.95	1.05	0.97,1.13	1.16	1.08,1.25
Black African	0.63	0.59,0.68	0.63	0.59,0.68	0.74	0.69,0.80	0.81	0.75,0.87
Black Caribbean	0.31	0.27,0.35	0.31	0.28,0.35	0.34	0.30,0.38	0.38	0.33,0.43
Other Black	0.44	0.40,0.48	0.44	0.40,0.48	0.51	0.46,0.55	0.55	0.50,0.60
**Number of children in the household[4]**								
1 (reference)	1				1		1	
2	0.88	0.84,0.91			0.83	0.80,0.87	0.83	0.79,0.87
3	0.72	0.69,0.75			0.66	0.62,0.69	0.67	0.63,0.70
4 or more	0.47	0.45,0.48			0.44	0.41,0.46	0.45	0.43,0.48
**Household composition[5]**								
Adults with child(ren) (reference)	1				1		1	
Single adult with child(ren)	0.83	0.80,0.86			0.79	0.75,0.82	0.79	0.75,0.83
Other	1.03	0.98,1.08			0.90	0.85,0.96	0.90	0.85,0.96
**IDACI quintile[6]**								
1 - most deprived (reference)	1						1	
2	0.88	0.85,0.91					0.92	0.88,0.96
3	1.04	0.99,1.08					1.08	1.02,1.14
4	1.03	0.97,1.10					1.17	1.07,1.27
5 - least deprived	2.04	1.79,2.33					1.77	1.50,2.08
**Local authority**								
Barking	0.82	0.78,0.86					0.88	0.83,0.94
City & Hackney	0.52	0.49,0.54					0.64	0.60,0.68
Havering	1.46	1.38,1.55					1.33	1.23,1.43
Newham (reference)	1						1	
Redbridge	0.85	0.81,0.89					0.71	0.66,0.75
Tower Hamlets	1.46	1.38,1.55					1.17	1.08,1.27
Waltham Forest	0.92	0.88,0.97					0.93	0.87,0.99

[1]Odds ratio. [2]Confidence interval. [3]General practice. [4]Number of people aged 0-17.9 years sharing the same address on the date of the child's second birthday. [5]Household composition defined on the date of the child's second birthday. [6]2019 Income Deprivation Affecting Children Index quintile.

Sensitivity analysis 2: Analyses on a sub-sample of 109,711 children born between 1st January 2014 and 20th March 2018 and therefore eligible to receive their first MMR vaccination between 12 and 24 months of age between 1st January 2015 and 20th March 2020. The receipt of MMR vaccination among these children is expected to be unaffected by the 2019 Coronavirus (COVID-19) pandemic

Table 8: Pre-Coronavirus pandemic sample characteristics

	All children		
	**n**	**%**	**95% CI[1]**
**MMR[2] status**			
No MMR between 12 and 24 months	15647	14.3	14.1,14.5
MMR between 12 and 24 months	94064	85.7	85.5,85.9
**Number of GP-recorded addresses[3]**			
1	84940	77.4	77.2,77.7
2	23329	21.3	21.0,21.5
3 or more	1442	1.3	1.2,1.4
**Sex**			
Male	55847	50.9	50.6,51.2
Female	53864	49.1	48.8,49.4
**Ethnic background**			
White British	20668	18.8	18.6,19.1
White Irish	226	0.2	0.2,0.2
Other White	13571	12.4	12.2,12.6
Chinese	708	0.6	0.6,0.7
White & Asian	1163	1.1	1.0,1.1
White & Black African	834	0.8	0.7,0.8
White & Black Caribbean	866	0.8	0.7,0.8
Other Asian	3404	3.1	3.0,3.2
Other Mixed	2331	2.1	2.0,2.2
Other	4637	4.2	4.1,4.3
Bangladeshi	10703	9.8	9.6,9.9
Indian	6100	5.6	5.4,5.7
Pakistani	7570	6.9	6.8,7.1
Black African	5466	5.0	4.9,5.1
Black Caribbean	1086	1.0	0.9,1.1
Other Black	2977	2.7	2.6,2.8
Missing	27401	25.0	24.7,25.2
**Number of children in the household[4]**			
1	34501	31.4	31.2,31.7
2	35015	31.9	31.6,32.2
3	20454	18.6	18.4,18.9
4 or more	19741	18.0	17.8,18.2
**Household composition[5]**			
Adults with child(ren)	81167	74.0	73.7,74.2
Single adult with child(ren)	18521	16.9	16.7,17.1
Other	10023	9.1	9.0,9.3
**IDACI quintile[6]**			
1 - most deprived	45041	41.1	40.8,41.3
2	40686	37.1	36.8,37.4
3	15544	14.2	14.0,14.4
4	6069	5.5	5.4,5.7
5 - least deprived	2346	2.1	2.1,2.2
Missing	25	0.0	0.0,0.0

Continued

Table 8: Continued

**Local authority**			
Barking	14089	12.8	12.6,13.0
City & Hackney	14201	12.9	12.7,13.1
Havering	13228	12.1	11.9,12.3
Newham	19190	17.5	17.3,17.7
Redbridge	17566	16.0	15.8,16.2
Tower Hamlets	15353	14.0	13.8,14.2
Waltham Forest	16084	14.7	14.5,14.9

[1]Confidence interval. [2]Residential anonymised linkage fields. [3]Number of people aged 0-17.9 years sharing the same RALF on the date of the child's second birthday. [4]Household composition defined on the date of the child's second birthday. [5]2019 Income Deprivation Affecting Children Index quintile. [6]Measles, mumps and rubella vaccination status. [7]No measles, mumps and rubella (MMR) vaccination recorded in the child's electronic health record between 12 and 24 months of age.

Table 9: Pre-Coronavirus pandemic sub-sample characteristics by first measles, mumps and rubella vaccination status

	No MMR[1]			MMR[2]		
	n	%	95% CI[3]	n	%	95% CI[3]
**All**	15647	14.3	14.1,14.5	94064	85.7	85.5,85.9
**Number of RALFs[4]**						
1	9804	11.5	11.3,11.8	75136	88.5	88.2,88.7
2	5436	23.3	22.8,23.8	17893	76.7	76.2,77.2
3 or more	407	28.2	26.0,30.6	1035	71.8	69.4,74.0
**Sex**						
Male	8089	14.5	14.2,14.8	47758	85.5	85.2,85.8
Female	7558	14.0	13.7,14.3	46306	86.0	85.7,86.3
**Ethnic background**						
White British	2114	10.2	9.8,10.6	18554	89.8	89.4,90.2
White Irish	52	23.0	18.0,28.9	174	77.0	71.1,82.0
Other White	2643	19.5	18.8,20.2	10928	80.5	79.8,81.2
Chinese	57	8.1	6.3,10.3	651	91.9	89.7,93.7
White & Asian	120	10.3	8.7,12.2	1043	89.7	87.8,91.3
White & Black African	119	14.3	12.1,16.8	715	85.7	83.2,87.9
White & Black Caribbean	162	18.7	16.2,21.4	704	81.3	78.6,83.8
Other Asian	357	10.5	9.5,11.6	3047	89.5	88.4,90.5
Other Mixed	349	15.0	13.6,16.5	1982	85.0	83.5,86.4
Other	1165	25.1	23.9,26.4	3472	74.9	73.6,76.1
Bangladeshi	864	8.1	7.6,8.6	9839	91.9	91.4,92.4
Indian	703	11.5	10.7,12.4	5397	88.5	87.6,89.3
Pakistani	867	11.5	10.8,12.2	6703	88.5	87.8,89.2
Black African	768	14.1	13.2,15.0	4698	85.9	85.0,86.8
Black Caribbean	267	24.6	22.1,27.2	819	75.4	72.8,77.9
Other Black	560	18.8	17.4,20.3	2417	81.2	79.7,82.6
Missing	4480	16.3	15.9,16.8	22921	83.7	83.2,84.1
**Number of children in the household[5]**						
1	3976	11.5	11.2,11.9	30525	88.5	88.1,88.8
2	4440	12.7	12.3,13.0	30575	87.3	87.0,87.7
3	3042	14.9	14.4,15.4	17412	85.1	84.6,85.6
4 or more	4189	21.2	20.7,21.8	15552	78.8	78.2,79.3
**Household composition[6]**						
Adults with child(ren)	11341	14.0	13.7,14.2	69826	86.0	85.8,86.3
Single adult with child(ren)	2965	16.0	15.5,16.5	15556	84.0	83.5,84.5
Other	1341	13.4	12.7,14.1	8682	86.6	85.9,87.3
**IDACI quintile[7]**						
1 - most deprived	6158	13.7	13.4,14.0	38883	86.3	86.0,86.6
2	6361	15.6	15.3,16.0	34325	84.4	84.0,84.7
3	2116	13.6	13.1,14.2	13428	86.4	85.8,86.9
4	813	13.4	12.6,14.3	5256	86.6	85.7,87.4
5 - least deprived	189	8.1	7.0,9.2	2157	91.9	90.8,93.0
Missing	10	40.0	23.0,59.7	15	60.0	40.3,77.0
**Local authority**						
Barking	2138	15.2	14.6,15.8	11951	84.8	84.2,85.4
City & Hackney	3149	22.2	21.5,22.9	11052	77.8	77.1,78.5
Havering	1251	9.5	9.0,10.0	11977	90.5	90.0,91.0
Newham	2601	13.6	13.1,14.0	16589	86.4	86.0,86.9
Redbridge	2797	15.9	15.4,16.5	14769	84.1	83.5,84.6
Tower Hamlets	1378	9.0	8.5,9.4	13975	91.0	90.6,91.5
Waltham Forest	2333	14.5	14.0,15.1	13751	85.5	84.9,86.0

[1]No measles, mumps and rubella (MMR) vaccination recorded in the child's electronic health record between 12 and 24 months of age. [2]MMR vaccination recorded in the child's electronic health record between 12 and 24 months of age. [3]Confidence interval. [4]Residential anonymised linkage fields (RALFs). [5]Number of people aged 0-17.9 years sharing the same RALF on the date of the child's second birthday. [6]Household composition defined on the date of the child's second birthday. [7]2019 Income Deprivation Affecting Children Index quintile.

Table 10: Binary logistic regression models estimating odds ratios for measles, mumps and rubella vaccination by 24 months of age

	Univariable		Number of GP-recorded addresses + individual-level characteristics		Number of GP-recorded addresses + individual- and household-level characteristics		Number of GP-recorded addresses + individual-, household-, and area-level characteristics	
	OR[1]	95% CI[2]	OR[1]	95% CI[2]	OR[1]	95% CI[2]	OR[1]	95% CI[2]
**Number of GP-recorded addresses[3]**								
1 (reference)	1		1		1		1	
2	0.43	0.41,0.45	0.46	0.44,0.48	0.46	0.44,0.48	0.45	0.43,0.47
3 or more	0.33	0.30,0.37	0.34	0.29,0.38	0.34	0.30,0.39	0.33	0.29,0.38
**Sex**								
Male (reference)	1		1		1		1	
Female	1.04	1.00,1.07	1.03	0.99,1.07	1.03	0.99,1.07	1.03	0.99,1.07
**Ethnic background**								
White British (reference)	1		1		1		1	
White Irish	0.38	0.28,0.52	0.42	0.31,0.58	0.44	0.32,0.61	0.48	0.35,0.67
Other White	0.47	0.44,0.50	0.5	0.47,0.54	0.50	0.47,0.54	0.55	0.51,0.59
Chinese	1.30	0.99,1.71	1.38	1.05,1.82	1.31	0.99,1.73	1.35	1.02,1.78
White & Asian	0.99	0.82,1.20	1.01	0.83,1.22	0.99	0.81,1.20	1.06	0.87,1.30
White & Black African	0.68	0.56,0.84	0.72	0.59,0.88	0.77	0.63,0.94	0.83	0.68,1.02
White & Black Caribbean	0.50	0.41,0.59	0.51	0.43,0.61	0.53	0.44,0.63	0.57	0.48,0.68
Other Asian	0.97	0.86,1.09	0.97	0.86,1.10	1.05	0.93,1.18	1.16	1.02,1.31
Other Mixed	0.65	0.57,0.73	0.67	0.59,0.76	0.68	0.60,0.77	0.74	0.66,0.84
Other	0.34	0.31,0.37	0.36	0.33,0.39	0.43	0.39,0.46	0.54	0.49,0.59
Bangladeshi	1.30	1.19,1.41	1.31	1.21,1.43	1.49	1.37,1.62	1.43	1.30,1.56
Indian	0.87	0.80,0.96	0.94	0.86,1.03	0.94	0.86,1.04	1.08	0.98,1.19
Pakistani	0.88	0.81,0.96	0.90	0.83,0.98	1.05	0.97,1.15	1.21	1.10,1.32
Black African	0.70	0.64,0.76	0.73	0.67,0.80	0.84	0.77,0.92	0.92	0.84,1.01
Black Caribbean	0.35	0.30,0.40	0.36	0.31,0.42	0.39	0.34,0.45	0.44	0.38,0.52
Other Black	0.49	0.44,0.54	0.51	0.46,0.56	0.58	0.52,0.65	0.64	0.57,0.71
**Number of children in the household[4]**								
1 (reference)	1				1		1	
2	0.90	0.86,0.94			0.84	0.80,0.89	0.84	0.79,0.89
3	0.75	0.71,0.78			0.67	0.63,0.71	0.67	0.63,0.71
4 or more	0.48	0.46,0.51			0.46	0.43,0.48	0.47	0.45,0.50
**Household composition[5]**								
Adults with child(ren) (reference)	1				1		1	
Single adult with child(ren)	0.85	0.82,0.89			0.84	0.79,0.88	0.83	0.79,0.88
Other	1.05	0.99,1.12			0.90	0.83,0.97	0.90	0.83,0.97
**IDACI quintile[6]**								
1 - most deprived (reference)	1						1	
2	0.85	0.82,0.89					0.92	0.88,0.97
3	1.01	0.95,1.06					1.08	1.01,1.16
4	1.02	0.95,1.11					1.16	1.04,1.28
5 - least deprived	1.81	1.55,2.10					1.66	1.37,2.01
**Local authority**								
Barking	0.88	0.82,0.93					1.00	0.92,1.07
City & Hackney	0.55	0.52,0.58					0.65	0.61,0.70
Havering	1.50	1.40,1.61					1.49	1.36,1.64
Newham (reference)	1						1	
Redbridge	0.83	0.78,0.88					0.71	0.66,0.76
Tower Hamlets	1.59	1.48,1.70					1.28	1.16,1.41
Waltham Forest	0.92	0.87,0.98					0.91	0.85,0.98

[1]Odds ratio. [2]Confidence interval. [3]General practice. [4]Number of people aged 0-17.9 years sharing the same address on the date of the child's second birthday. [5]Household composition defined on the date of the child's second birthday. [6]2019 Income Deprivation Affecting Children Index quintile.

Table 11: Attributable risk percentage[1]

	No MMR[2] between 12 and 24 months	MMR[2] between 12 and 24 months	Total
**Number of GP-recorded addresses[3]**			
2 or more	8410 (a)	25383 (b)	33793 (a+b)
1	14581 (c)	102575 (d)	117156 (c+d)
**Total**	22991 (a+c)	127958 (b+d)	150949 (N)

[1]Population attributable risk (PAR)=(overall risk–risk among unexposed =((a+c)/N)-(c/(c+d)) = (22991/150949)-(14581/117156)=0.028. Population attributable risk percentage (PAR%)=PAR/overall risk=((a+c/N)-(c/(c+d)))/((a+c)/N)= 0.028/(22991/150949)=18.4%. [2]Measles, mumps and rubella vaccination. [3]General practice.
